# Therapeutic targeting of anoikis resistance in cutaneous melanoma metastasis

**DOI:** 10.3389/fcell.2023.1183328

**Published:** 2023-04-26

**Authors:** Hannah M. Neuendorf, Jacinta L. Simmons, Glen M. Boyle

**Affiliations:** ^1^ Cancer Drug Mechanisms Group, QIMR Berghofer Medical Research Institute, Herston, QLD, Australia; ^2^ School of Biomedical Sciences, Faculty of Health, Queensland University of Technology, Brisbane, QLD, Australia; ^3^ School of Biomedical Sciences, Faculty of Medicine, University of Queensland, Brisbane, QLD, Australia

**Keywords:** melanoma, anoikis, therapy, metastasis, repurposing, targeting

## Abstract

The acquisition of resistance to anoikis, the cell death induced by loss of adhesion to the extracellular matrix, is an absolute requirement for the survival of disseminating and circulating tumour cells (CTCs), and for the seeding of metastatic lesions. In melanoma, a range of intracellular signalling cascades have been identified as potential drivers of anoikis resistance, however a full understanding of the process is yet to be attained. Mechanisms of anoikis resistance pose an attractive target for the therapeutic treatment of disseminating and circulating melanoma cells. This review explores the range of small molecule, peptide and antibody inhibitors targeting molecules involved in anoikis resistance in melanoma, and may be repurposed to prevent metastatic melanoma prior to its initiation, potentially improving the prognosis for patients.

## 1 Introduction

Anoikis is a type of apoptosis induced by detachment from the extracellular matrix (ECM) and surrounding cells (Meredith et al., 1993; Frisch and Francis, 1994). Under a physiological state, anoikis functions to maintain cell number equilibrium by triggering apoptosis in cells with inappropriate cell-to-ECM and cell-to-cell interactions (Frisch and Francis, 1994). Anoikis thereby functions in preventing cells from migrating, and subsequently proliferating at inappropriate sites within the body. However, as a hallmark of cancer and an absolute requirement for metastasis, tumour cells must acquire mechanisms to resist cell death by anoikis to detach from the primary lesion, persist in circulation and facilitate the seeding of metastases (Hanahan, 2022). In these cells, anoikis resistance is achieved through the oncogenic deregulation of survival and death signalling as a result of genetic, epigenetic and (micro-) environmental variation.

**FIGURE 1:**
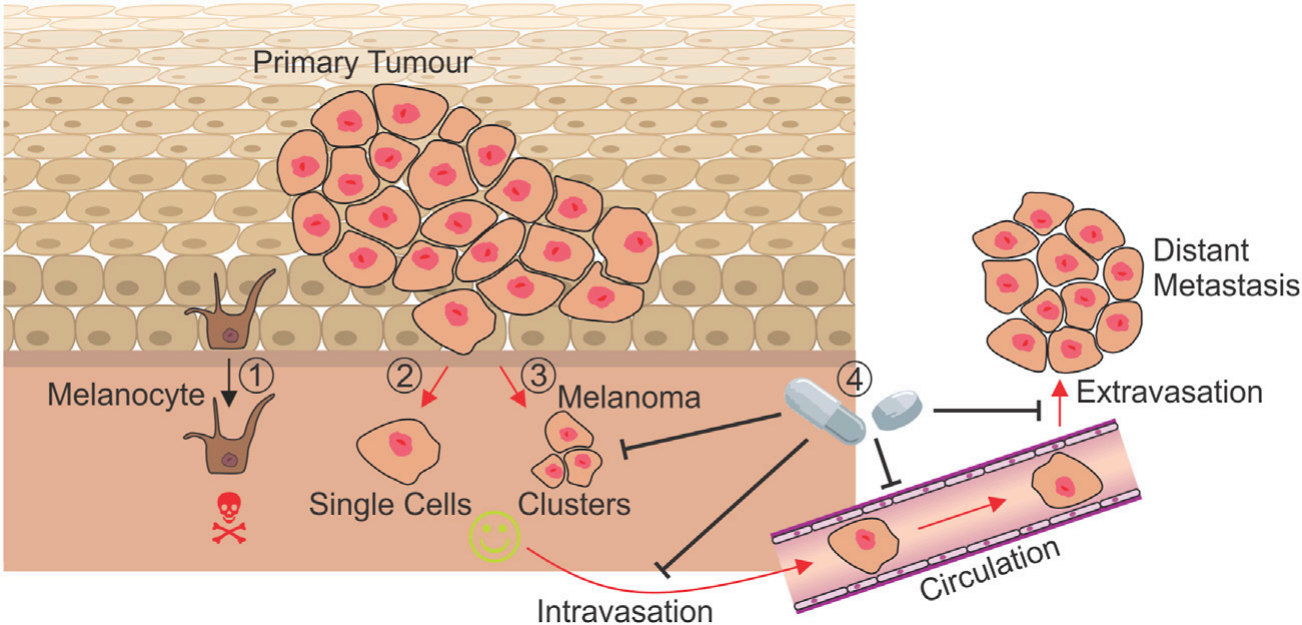
Mechanisms of anoikis resistance during metastasis. While the detachment of a non-transformed melanocyte results in cell death by anoikis (1), melanoma cells leaving the dermis as either single cells (2) or clusters (3) must develop mechanisms to resist anoikis in order to metastasise and form secondary tumours at distant sites. Treatments targeting currently known mechanisms of anoikis resistance have the potential to prevent cells leaving the primary tumour (4), and may target those in circulation, preventing the seeding of metastases and improving prognosis for patients. Red arrows: Processes where anoikis resistance is absolutely required.

Cutaneous Malignant Melanoma, a cancer arising from melanocytes in the skin, is a highly aggressive and invasive cancer type that demonstrates early dissemination into lymphatic circulation from primary tumours less than 0.5 mm thick in at least one-third of patients (Werner-Klein et al., 2018). In addition, metastatic melanoma is a vastly complex and heterogeneous disease, with multiple, highly plastic subpopulations of cells contributing to disease progression and drug resistance (Tsoi et al., 2018; Rambow et al., 2019). Despite significant advancement in the treatment of metastatic melanoma since the advent of targeted and immune checkpoint inhibitors, three-quarters of patients who are diagnosed or relapse with an advanced stage of melanoma inevitably succumb to the disease (AIHW, 2021). With the incidence of melanoma predicted to increase into the future (Guy et al., 2015; Whiteman et al., 2016), the development or repurposing of existing drugs to target and prevent the progression of melanoma are urgently required. Anoikis resistance poses an attractive target for the therapeutic intervention of melanoma progression, with potential to prevent the dissemination of cells from the primary tumour and allow the targeting of circulating tumour cells (CTCs). However, the understanding of mechanisms involved in anoikis resistance in melanoma remains limited. This review details anoikis resistance mechanisms identified in cutaneous melanoma, examines therapeutic inhibitors with potential to be repurposed to target anoikis resistance mechanisms, and discusses the feasibility of targeting anoikis resistance to prevent the progression of melanoma to metastasis (Figure 1).

### 1.1 General mechanisms of anoikis resistance

Anoikis occurs through a combination of both the intrinsic and extrinsic apoptosis pathways converging at the mitochondria and resulting in the activation of caspases, triggering DNA fragmentation and cell death (Paoli et al., 2013). The Bcl-2 family of proteins play a key role in both apoptosis pathways and is comprised of three subgroups: the anti-apoptotic proteins (Bcl-2, Bcl-X_L_, Bcl-w, Mcl-1 and A1) (Opferman and Kothari, 2018), the multi-domain pro-apoptotic members (Bax, Bak, Bok) (Reed, 2006), and the BH3-only pro-apoptotic proteins (Bim, Bad, Bid, Noxa, Puma) (Adams and Cory, 2007; Hao et al., 2007; Anvekar et al., 2011). Following cellular detachment, the pro-apoptotic members translocate from the cytosol to the mitochondria where they cause mitochondrial outer-membrane permeabilisation (MOMP) and cytochrome *c* release, triggering apoptosome assembly, caspase cleavage and cell death (Gilmore, 2005). As a result, it’s generally accepted that anoikis is marked by activation of caspase-3, -8 and -9, and PARP-1 cleavage upon anchorage loss (Zhang et al., 2011; Hasnat et al., 2015; Fanfone et al., 2022). However in the case of malignant cells, activation of oncogenic signalling results in deregulation of the pathways triggering caspase activation and PARP cleavage, inevitably resulting in abrogated apoptosis despite the presence of cell death-inducing stimuli.

**FIGURE 2:**
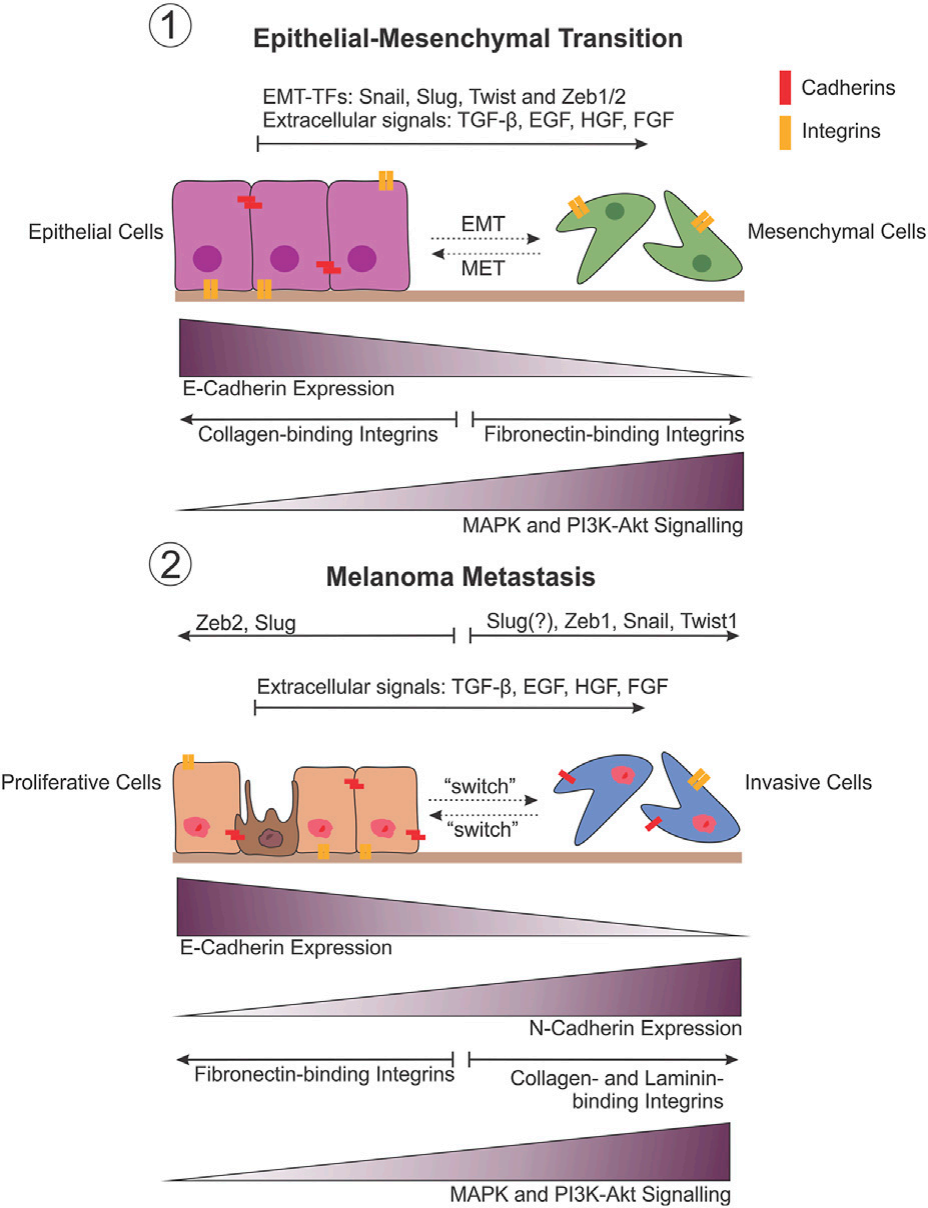
Comparison between EMT and melanoma metastasis. Phenotypic plasticity observed during melanoma metastasis draws striking similarities with the changes that occur during epithelial-mesenchymal transition (EMT) (1). This is believed to be the result of the developmental lineage of melanocytes in the neural crest, allowing melanoma cells to undergo an EMT-like transition to facilitate metastasis by varying the expression of adhesion molecules in particular, in response to shifts in transcriptional regulation (EMT-TFs) (2).

Importantly, the “attach or die” phenotype is not true for all cells, and applies mostly to those of epithelial origin, while circulating blood and immune cells, for example, are intrinsically anoikis resistant (Zhu et al., 2001). As an exception to this rule, epithelial cells are able to overcome anoikis under certain conditions such as embryogenesis and wound healing, through the process of epithelial-to-mesenchymal transition (EMT). EMT allows epithelial cells to gain reversible migratory and invasive properties, as well as stem cell-ness and the ability to evade apoptosis with the loss of cell-to-cell adhesion in response to microenvironmental cues (Kalluri and Weinberg, 2009; Thiery et al., 2009). Despite residing in the skin, melanocytes are not derived from the epithelial lineage and therefore do not undergo true EMT (Kalluri and Weinberg, 2009). Rather, EMT is involved in the formation of melanoblasts (Figure 2, melanocyte precursor cells) from neural crest cells during embryonic development (Thomas and Erickson, 2008; Kalluri and Weinberg, 2009; Vandamme and Berx, 2014). As a result of their developmental lineage, melanoma cells are able to “hijack” EMT-like pathways to facilitate metastatic progression, including driving resistance to anoikis (Alonso et al., 2007). Subsequently, a model has been proposed that describes the intrinsic phenotypic plasticity observed in distinct subpopulations of melanoma cells; the phenotype switching model. Similar to the way epithelial cells are able to reversibly switch between epithelial and mesenchymal phenotypes, this model describes the ability of cells in the primary tumour to switch between proliferative and invasive transcriptional states subject to regulation by the proximal microenvironment (Hoek et al., 2006; Hoek et al., 2008). Subsequently, many of the molecules identified as drivers of EMT have likewise been identified to contribute to the acquisition of anoikis resistance in melanoma (Figure 2).

## 2 Mechanisms of anoikis resistance and potential therapies

### 2.1 Adhesion molecules

#### 2.1.1 Integrins

Integrins are transmembrane cell surface receptors that are primarily responsible for mediating cell adhesion to the ECM (Paoli et al., 2013). In addition to their function in physical attachment, integrins transduce signals from the ECM to the inner machinery of the cell by directly binding components of the cytoskeleton. This results in actin filament rearrangement within the cell and activation of downstream signalling pathways that promote migration, proliferation and survival. In addition to their role in “outside-in” signalling, integrins can transduce “inside-out” signalling via the activation of extra-cellular ligands, particularly TGF-β (Barczyk et al., 2010). Integrins consist of two transmembrane glycoprotein subunits, α and β, which associate via non-covalent bonding interactions (Hynes, 2002). Depending on the cell type and composition of the ECM, different types of integrins may be present; those that bind fibronectin, laminin, vitronectin, or collagen (Friedl and Wolf, 2003).

During the process of EMT, epithelial cells undergo the phenomenon of ‘integrin switching’, where collagen-binding integrins are downregulated, and fibronectin-binding integrins upregulated to allow detachment from the basement membrane and mesenchymal cell migration (Maschler et al., 2005). As a hallmark of cancer, self-sufficiency in growth signalling is an absolute requirement for disease progression. A prominent mechanism employed by cancer cells to achieve this is integrin switching, as it favours receptors that transmit pro-growth or pro-survival signals, including those that prevent cell death upon detachment. As such, a distinct change in integrin expression has been observed between melanocytes and melanoma cells throughout progression. Studies analysing integrin expression demonstrate that melanocytes predominantly express the α3β1 and α6β1 laminin-binding integrins for adhesion to the basement membrane *in vivo* (Krengel et al., 2005). However, during the progression to malignant disease, melanoma cells upregulate expression of fibronectin- and collagen-binding integrins to facilitate vertical migration through the dermis, and attachment to vascular endothelial cells allowing dissemination into circulation.

To date, the most comprehensively studied integrin with links to anoikis resistance in melanoma metastasis is the αvβ3 integrin. Studies demonstrate αvβ3 binds multiple ECM components, including fibronectin, vitronectin, fibrinogen and osteopontin (Cheresh, 1987). It is best known for its role in angiogenesis, where blocking of the αvβ3 receptor in melanoma patients through the administration of combination high-dose tumour necrosis factor (TNF) and interferon γ (IFN-γ) inhibits tumour angiogenesis and growth due to the specific inhibitory effect of these cytokines on β3 subunit protein synthesis (Defilippi et al., 1991; Brooks et al., 1994; Rüegg et al., 1998). In addition, expression of αvβ3 on the surface of invasive cells allows the recruitment, localisation and activation of matrix metalloproteinases (MMP-1 and MMP-2), facilitating ECM remodelling through collagen degradation, promoting migration (Brooks et al., 1996; Brooks et al., 1998; Hofmann et al., 2000). As such, αvβ3 integrin is widely recognised as a molecular marker of metastasis, where its expression correlates with melanoma progression from radial growth phase (RGP) to the invasive vertical growth phase (VGP) (Albelda et al., 1990; Danen et al., 1994; Hsu et al., 1998; Johnson, 1999; Van Belle et al., 1999; Kageshita et al., 2000; Alonso et al., 2007). Expression of αvβ3 integrin in primary cutaneous melanoma is likewise associated with increased sentinel lymph node metastasis (Meves et al., 2015).

Importantly, αvβ3 integrin has been demonstrated to contribute to anoikis resistance through driving upregulation of anti-apoptotic proteins such as Bcl-2 (Montgomery et al., 1994; Petitclerc et al., 1999). A five-fold increase in the relative Bcl-2/Bax ratio conferred increased cellular survival (Petitclerc et al., 1999; Zhang et al., 2011). Recent studies have demonstrated that the αvβ3 integrin is able to induce partial EMT independent of TGF-β signalling (Kariya et al., 2021). This is important considering the recent emergence of evidence for distinct partial EMT-like phenotypes in melanoma that possess increased invasiveness and motility (Sahoo et al., 2022). However, an independent study demonstrated that αvβ3 integrin expression was not influenced by Snail, a transcription factor that promotes EMT (Kuphal et al., 2005).

Integrins containing the β1-subunit in conjunction with a range of alpha subunits (α2, α3, α4, α5, α6 or α7) have similarly been implicated in melanoma metastasis, invasion and anoikis resistance (Etoh et al., 1993; Danen et al., 1994; Ziober et al., 1999; Hegerfeldt et al., 2002; Toricelli et al., 2013; Kolli-Bouhafs et al., 2014; Kozlova et al., 2018; Kozlova et al., 2019; Kozlova et al., 2020). Kozlova and others demonstrated that inhibition of integrin α2β1 increased sensitivity to anoikis, while blocking α3β1 and α5β1 reduced invasion and activation of MMP-2 *in vitro* (Kozlova et al., 2019; Kozlova et al., 2020). Integrins containing the β1 subunit are thought to confer increased cell survival under anchorage-independent conditions through increased expression of Bcl-2 and c-Myc (Kozlova et al., 2019).

**FIGURE 3:**
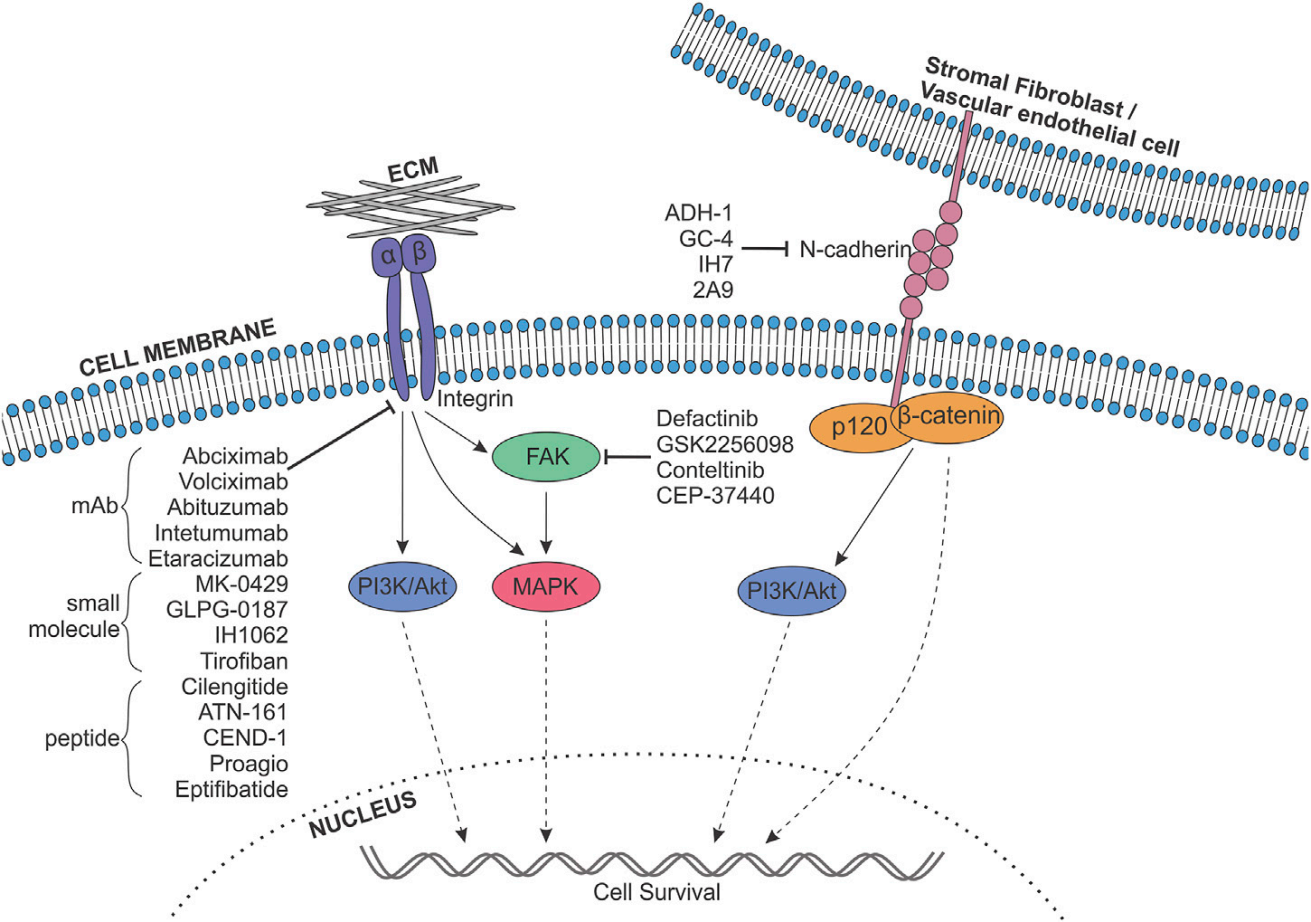
Targeting adhesion molecules in melanoma anoikis resistance. A range of integrins, as well as N-cadherin, and their downstream signalling pathways, including FAK (green), Mitogen-Activated Protein Kinase (MAPK; pink), PI3K-Akt (purple) and catenin pathways (orange), are known to contribute to anoikis resistance in melanoma. Pharmaceutical inhibitors targeting integrins, cadherins and FAK exist, and have the potential to be repurposed to block melanoma cell survival upon detachment. Different classes of integrin inhibitors are shown, mAb, monoclonal antibody; ECM, Extracellular matrix. Indicated inhibitors have been sourced from data from all cancers.

Integrins play an important role in the progression of melanoma, however data surrounding the activation and deactivation of integrin expression and clustering throughout the metastatic process, remains incomplete. Studies investigating integrins in metastasis focus on their expression levels and largely ignore their activity. It’s understood that integrin internalisation through endocytosis is important in enabling cell motility as it allows their recycling to the leading edge of migrating cells (Krengel et al., 2005). Therefore, future studies investigating integrin recycling throughout each stage of metastasis would provide valuable information on their functional role. Nevertheless, inhibitors targeting integrin clustering and expression have been used successfully in a range of non-malignant diseases (Slack et al., 2022). Given that integrins are strongly upregulated in a range of cancers including melanoma, the use of integrin inhibitors in this context has been extensively explored (Figure 3).

Eptifibatide (integrilin) and tirofiban (aggrastat) target β3 integrins, and were registered by the FDA (1998) for use in patients with heart conditions. While neither inhibitor has been examined in clinical trials against any cancer type, Kim et al. demonstrated that eptifibatide significantly reduced the ability of B16-F10 melanoma cells to form nodules on the lungs of mice following arsenic exposure (Kim et al., 2019).

A number of monoclonal antibody therapies targeting integrins have been evaluated in clinical trials (Table 1). However, none of these therapies have progressed past phase II trials. In addition, many small molecule and peptide compounds have demonstrated efficacy as integrin inhibitors. Cilengitide is a cyclic Arg-Gly-Asp (iRGD) peptide that inhibits αvβ3 and αvβ5 integrins, thus inhibiting tumour cell interactions with vascular endothelial cells, as well as cell-matrix interactions and angiogenesis. A phase III clinical trial examining its use in glioblastoma was completed (NCT00689221), in addition to multiple phase II studies in a range of cancers (NCT00093964, NCT00813943, NCT01124240, NCT00103337, NCT00121238, NCT00842712, NCT00679354). Despite preclinical evidence supporting its use in melanoma patients (Lode et al., 1999), a phase II trial in stage III/IV metastatic melanoma patients was terminated (NCT00082875) due to low Progression Free Survival (PFS) after 8 weeks, indicating the treatment was not effective (Kim et al., 2012). Nevertheless, more recent studies revealed the ability of cilengitide to inhibit invasion and vasculogenic mimicry of melanoma cells, and inhibit adhesion to vitronectin (Ruffini et al., 2015) indicating that further investigation into the use of cilengitide in metastatic melanoma patients may be warranted. While one study advised against the use of cilengitide in combination with paclitaxel due to decreased sensitivity to the chemotherapeutic agent (Stojanović et al., 2018), another revealed that combination therapy with doxorubicin synergistically suppressed tumour growth and reversed drug resistance *in vivo*, extending the survival of mice subcutaneously injected with A375 cells expressing β3 integrin (Zhu X. et al., 2019). Furthermore, combining cilengitide with anti-PD-L1 therapy significantly reduced tumour volume in a B16 murine melanoma model, and positively regulated anti-tumour immune responses (Pan et al., 2022).

**TABLE 1:** Monoclonal antibody therapies targeting integrins.

Inhibitor	FDA registration year/trial phase	Clinical trial no.	Disease types	Evidence in melanoma
Abciximab	1993 (FDA)		Cardiac Ischemic Complications	Varner et al. (1999), Trikha et al. (2002)
Volociximab	Phase I	NCT00654758, NCT00666692	Non-small Cell Lung Cancer	Bhaskar et al. (2007), Ricart et al. (2008)
	Phase II	NCT00635193, NCT00516841	Ovarian Cancer	
	Phase II	NCT00100685	Renal Cell Carcinoma	
	Phase II	NCT00401570	Pancreatic Cancer	
	Phase II	NCT00278187	Lung Cancer	
	Phase II	NCT00369395, NCT00099970	Melanoma	
Abituzumab	Phase I	NCT00848510	Colorectal and Ovarian Cancer Patients with Liver Metastases	Castel et al. (2000), Mitjans et al. (2000), Wagner et al. (2010)
	Phase II	NCT01008475	Metastatic Colorectal Cancer	
Intetumumab	Phase I/II	NCT00246012	Stage IV melanoma	Trikha et al. (2004), Wu et al. (2017b), O'Day et al. (2011), O'Day et al. (2012), Robinson et al. (2012)
Etaracizumab (Abegrin)	Phase I and II	NCT00111696, NCT00066196	Metastatic Melanoma	Posey et al. (2001), Hersey et al. (2010), Moschos et al. (2010)
	Phase II	NCT00072930	Prostate Cancer	
	Phase I/II	NCT00263783, NCT00284817	Refractory Solid Tumours	

A small peptide antagonist ATN-161, similarly targeting αvβ3 and αvβ5 integrins, has been tested in phase I/II trials with carboplatin (NCT00352313). Preclinical evidence utilising ATN-161 loaded reversibly cross-linked polymersomes for drug delivery into C57BL/6 mice bearing B16-F10 tumours demonstrated significant inhibition of tumour growth, and significantly improved survival rates (Zhang et al., 2017). Likewise, MK-0429 is a non-peptide antagonist compound initially examined in a phase II trial for the treatment of osteoporosis [NCT00533650 (Hutchinson et al., 2003)] for its ability to target the αvβ3 receptor. MK-0429 reduced the number of metastatic colonies in the lungs of mice injected with B16-F10 melanoma cells by 64% at 100 mg/kg (Pickarski et al., 2015). Similar to cilengitide, CEND-1 is an Arg-Gly-Asp cyclic peptide (iRGD) targeting αvβ3 and αvβ5 integrins in the tumour vasculature, promoting tumour penetration to enhance the efficacy and specificity of chemotherapy treatment. A phase I clinical trial in pancreatic ductal adenocarcinoma (PDAC) patients combining CEND-1 with nabpaclitaxel and gemcitabine was successfully completed [NCT03517176 (Dean et al., 2020)]. The use of CEND-1 in melanoma patients has not been examined in clinical trials. However preclinical evidence demonstrated that sterically-stabilised liposomes modified with iRGD peptides such as CEND-1, containing either paclitaxel or doxorubicin, significantly reduce volume of melanoma tumours and increase percentage survival in murine models (Yu et al., 2013; Du et al., 2014).

IH1062 is a novel small molecule inhibitor of αvβ3 integrins. Preclinical evidence in melanoma demonstrated its ability to induce anoikis and suppress metastasis in human melanoma cells, interrupting ECM attachment and FAK phosphorylation, and resulting in caspase activation through a decrease in the Bcl-2/Bax protein ratio (Zhang et al., 2011). Other molecules with therapeutic potential that are yet to be examined in melanoma are summarised in Table 2.

**TABLE 2:** Pharmacological Inhibitors yet to be examined in melanoma.

Target	Inhibitor	FDA registration year/trial phase	Clinical trial no.	Disease types
αvβ3, β1, β5, β6 and α5 integrins	GLPG-0187	Phase I	NCT01580644, NCT00928343, NCT01313598 (Cirkel et al., 2016)	Solid Tumours
αvβ3 integrins	Proagio	Phase I	NCT05085548, (Turaga et al., 2016; Turaga et al., 2021)	Pancreatic Cancer
		Preclinical		Uveal Melanoma and Triple Negative Breast Cancer (Yang et al., 2015; Sharma et al., 2021)
N-cadherin EC1-3 domain	IH7	Preclinical	N/A	Prostate Cancer (Tanaka et al., 2010)
N-cadherin EC4 domain	2A9	Preclinical	N/A	Prostate Cancer (Tanaka et al., 2010)
FAK	GSK2256098 (UNII-R7O0O4110G, CAS 1224887–10–8)	Phase I	NCT01138033, NCT01938443, NCT00996671	Solid Tumours
		Phase II	NCT02428270	Pancreatic Cancer, Adenocarcinoma
		Phase II	NCT02523014 (recruiting)	Progressive Meningioma
FAK	Conteltinib	Phase I	NCT02695550	Non-Small Cell Lung Cancer
FAK	CEP-37440	Phase I	NCT01922752	Solid Tumours
Akt	Miransertib (MK-7075)	Phase I	NCT02594215	*Proteus* Syndrome
		Phase I	NCT01473095	Advanced Solid Tumours, Lymphoma
Akt	TAS-117	Phase II	NCT03017521 (Lee et al., 2021)	Solid Tumours
Akt	LY-2780301	Phase I	NCT02018874, NCT01115751	Non-Hodgkin’s Lymphoma, Advanced Solid Tumours
		Phase I	NCT01115751	Metastases

Caution must be used when considering integrin-targeting inhibitors for the treatment of melanoma patients. Natalizumab, for example, targets α4, β7 and β1 integrins, and was registered by the FDA in 2004 for multiple sclerosis patients. However, multiple studies have reported a significantly increased risk of developing cutaneous melanomas following natalizumab treatment (Mullen et al., 2008; Vavricka et al., 2011; Munguía-Calzada et al., 2017; Sabol et al., 2017; Kelm et al., 2019). As α4β1 integrin expression is thought to prevent melanoma metastasis formation, therapeutic inhibition with natalizumab was shown to increase invasive potential and cell migration *in vitro*, and resulted in the dissolution of clusters to single cells (Qian et al., 1994; Carbone et al., 2020). These concerns are further compounded by the findings that targeting orthosteric binding sites of integrins has the potential to induce a shift in integrin binding affinity to a higher binding state, promoting tumour growth and angiogenesis rather than inhibiting it (Slack et al., 2022).

Furthermore, integrin therapies induce the detachment of melanoma cells from the ECM, but do not result in tumour cell apoptosis in most cases. Consequently, integrin inhibitors may induce amoeboid migration, characterised by a rounded cell morphology with bleb-like protrusions, and weak cell-ECM interactions (Wu J. S. et al., 2021), by encouraging detachment of melanoma cells from the primary tumour. For instance, in a study investigating the αvβ3 targeting cyclic oligopeptide cRGDfV, cell adhesion was blocked however apoptosis was not induced (Allman et al., 2000). Rather, a change in cell morphology imitating amoeboid migration was observed following treatment. A similar phenomenon was detected following the treatment of cells with abituzumab (Castel et al., 2000). Amoeboid invasion has been associated with weakening of integrin adhesions (Carragher et al., 2006; Wolf et al., 2007). While previously shown that β1 integrin expression is essential for amoeboid migration of melanoma cells (Sanz-Moreno et al., 2008), other studies have revealed that blocking β1 expression cannot abolish amoeboid crawling or dissemination (Hegerfeldt et al., 2002). To overcome this, combining integrin inhibitors with urokinase-plasminogen activator (uPAR) inhibitors may be effective in blocking amoeboid invasion as uPAR, in association with integrins and the actin cytoskeleton, are believed to drive amoeboid invasion (Margheri et al., 2014).

#### 2.1.2 Cadherins

Cadherins are calcium-dependent transmembrane glycoproteins that function in maintaining cell-to-cell adhesion through the formation of adherens junctions and contribute to intracellular signalling via p120 and β-catenin recruitment in the cytosol, influencing proliferation, cell survival and invasion (Venhuizen et al., 2020). There are several subtypes of cadherins, however type I cadherins are the most relevant to melanoma, and include: epithelial cadherin (E-cadherin, *CDH1*)*,* present in epithelial cells, melanocytes and keratinocytes; neural cadherin (N-cadherin, *CDH2*); and placental cadherin (P-cadherin, *CDH3*) (Yu et al., 2019).

In the skin, E-cadherin functions to maintain the adhesion of melanocytes to keratinocytes through the formation of the epidermal melanin unit; one melanocyte bound to ten keratinocytes (Tang et al., 1994; Danen et al., 1996; D'Arcy and Kiel, 2021). However, loss of E-cadherin is an early event in the progression of most melanomas, believed to occur between radial and vertical growth phases (Silye et al., 1998; Kuphal et al., 2004), and results in the loss of interactions within the epidermal melanin unit (Hsu M. et al., 2000). Coinciding with the reduced levels of E-cadherin, melanoma cells gain expression of N-cadherin allowing them to interact with stromal fibroblasts and endothelial cells, promoting migration from the epidermis and dissemination into circulation (Li et al., 2001a; Nguyen and Mège, 2016; Murtas et al., 2017). This ‘switch’ in cadherin expression is driven by downregulation of the EMT transcription factors (EMT-TFs) Snail and Zeb1 (Poser et al., 2001; Kuphal et al., 2004; Zhu L. et al., 2019) and correlates with a low proliferation rate and acquisition of an invasive phenotype in melanoma cells (Kovacs et al., 2016).

As overexpression of MITF is shown to increase E-cadherin expression, while directly repressing *CDH2* (N-cadherin), MITF downregulation results in cadherin switching in melanoma (Dilshat et al., 2021). Other molecules identified to regulate cadherin expression in melanoma cells include: STAT3 and MEK, demonstrated to induce expression of N-cadherin (Vultur et al., 2014); FAK, knockdown of which is shown to reduce expression of E-cadherin (Pei et al., 2017); and Integrin Linked Kinase (ILK) which regulates N-cadherin membranous localisation through regulating its’ endocytosis and recycling (Gil et al., 2020). Notch1 is likewise demonstrated to increase expression of N-cadherin in melanoma cells and coincides with the acquisition of an invasive phenotype (Murtas et al., 2017). Co-expression of these molecules within a patients’ lesion correlates with significantly poorer prognosis compared to those expressing either protein alone. Crucially, expression of N-cadherin facilitates metastasis by promoting the survival of cells under anchorage-independent conditions (Li et al., 2001a). This is achieved through the activation of anti-apoptotic proteins Akt and PKB, increased β-catenin levels, and the inactivation of pro-apoptotic protein Bad.

However, controversy arises between studies analysing the expression levels of E- and N-cadherin in *ex vivo* melanomas. Early studies indicated that the majority of melanoma tumours retain no expression of E-cadherin as a result of autocrine HGF secretion from melanoma cells activating c-Met and the MAPK and PI3K pathways resulting in E-cadherin downregulation (Li et al., 2001b). However, more recent studies demonstrated mixed expression of E- and N-cadherin in patient samples, trending towards an increase in N-cadherin expression as melanoma progresses (Yan et al., 2016). Granted, melanoma cells are known to be highly plastic in their protein expression and phenotype. Therefore, it’s possible that the E-to N-cadherin switch is highly transient and may occur only in a small subpopulation of cells with the propensity for metastasis, with E-cadherin re-expressed to promote adhesion and proliferation at the secondary tumour site. As a result the switch may not be identifiable through histological analysis of tumour sections. Furthermore, studies thus far fail to analyse the function and activity of the cadherins present. It is hypothesised that reduced activity, rather than reduced expression of E-cadherin may be sufficient to promote anoikis resistance in some patients. Nevertheless, the survival advantage conferred by cooperative dissemination and the formation of CTC clusters or circulating tumour microemboli (CTM) *versus* single cells suggests an important role for the expression of cell-to-cell adhesion molecules during metastasis and may explain why a partial switch in cadherin expression is observed in melanoma (Hou et al., 2011).

As a result of the switch in expression of E-to N-cadherin observed in a range of cancers driven by EMT-like transcriptional programs, therapies that promote a reversal of this transition have been utilised (Figure 3). ADH-1 (exherin) is a synthetic cyclic peptide containing the His-Ala-Val (HAV) sequence (N-Ac-CHAVC-NH2), designed to bind and inhibit N-cadherin clustering and interactions (Eslami et al., 2019), inhibiting angiogenesis and metastasis, and promoting apoptosis in multiple myeloma, neuroblastoma and pancreatic cancer (Shintani et al., 2008; Lammens et al., 2012; Sadler et al., 2013). Subsequently, the safety and efficacy of ADH-1 as a single-agent therapy against neoplasms has been examined in phase I/II trials (NCT00264433, NCT00265057), as well as in combination with carboplatin, docetaxel or capecitabine (NCT00390676), and melphalan (LPAM) (NCT00421811). In melanoma, pre-clinical evidence suggests combining systemic ADH-1 therapy with regionally infused LPAM has the potential to improve survival of melanoma patients with in-transit metastases. Studies investigating the combination in mouse models of melanoma demonstrate a synergistic reduction in tumour volume associated with suppression of N-cadherin expression, induction of apoptosis and changes in the levels of genes related to cell adhesion (Augustine et al., 2008; Turley et al., 2015). ADH-1 treatment further resulted in an increase in endothelial cell permeability, which was hypothesised to improve the delivery of chemotherapeutic agents to melanoma tumours. However, both studies demonstrate increased volume of specific tumours treated with ADH-1 alone, which appears to correlate with the PTEN expression status of the cell line employed. Additionally, examination of ADH-1 combined with temozolomide (TMZ) *in vivo* yielded conflicting results, demonstrating synergism in DM443 and DM366 xenograft models, but increased tumour volume in A375 xenografts. It is therefore clear that the mutation status of a patient is an important consideration for the administration of ADH-1 therapies. A phase I trial examining safety, pharmacokinetics and anti-tumour activity of ADH-1 + LPAM in stage IIIB/C melanoma patients with in-transit limb metastases demonstrated complete response in 50% of patients (N = 16) (Beasley et al., 2009), with a subsequent phase II study revealing the combination therapy resulted in significantly improved response rates when compared with standard-of-care isolated limb infusion (ILI) alone [NCT00421811 (Beasley et al., 2011)].

While yet to be examined in clinical trials, monoclonal antibody therapies designed to block N-cadherin activity have demonstrated preclinical efficacy in a range of cancers (Hazan et al., 2000; Zhang et al., 2007; Wallerand et al., 2010; Groen et al., 2011; Zhang et al., 2013; Eiring et al., 2015; Klymenko et al., 2017) (Table 2). GC-4 binds the EC1 domain of N-cadherin, blocking adhesion and intracellular signalling. Treatment of melanoma cells with GC-4 resulted in knockdown of N-cadherin, and subsequently blocked melanoma cell adhesion to endothelial cells, inhibiting transendothelial migration (Qi et al., 2005).

In addition to the use of therapies aimed at reducing N-cadherin activity, studies have investigated whether increasing E-cadherin expression within the primary tumour is a feasible method of reducing the loss of interactions within the epidermal melanin unit, thus preventing cells from entering circulation. Inducing expression of E-cadherin in melanoma cells is demonstrated to reduce colony formation, and restore keratinocyte-mediated inhibition of invasion, resulting in smaller tumours *in vivo* (Hsu M. Y. et al., 2000). Although no data has been presented to support this to date, it’s possible that the use of these agents may potentiate the attachment of CTCs to secondary sites that express E-cadherin, such as the liver, lungs and intestines, or may promote mesenchymal-to-epithelial transition (MET) of pre-existing micro-metastases resulting in E-cadherin re-expression, cellular proliferation and subsequent formation of clinically detectable metastases, as has been observed in breast cancer (Chao et al., 2010; Padmanaban et al., 2019).

### 2.2 Receptor tyrosine kinase (RTK) hyperactivation

Activated upon ligand binding or via ligand-independent mechanisms facilitated by integrins, tyrosine kinase receptors (RTKs) function as the upstream initiators of intracellular signalling cascades (Moro et al., 1998; Shen and Kramer, 2004; Mitra et al., 2011). Melanoma cells are demonstrated to significantly upregulate expression of a range of RTKs to maintain growth and survival signalling, avoid apoptosis and ultimately drive the processes of invasion and metastasis. RTK hyperactivation is also postulated to be one of the main mechanisms driving intrinsic and acquired resistance to treatment utilising targeted inhibitors against the MAPK signalling pathway (Manzano et al., 2016). In addition to the growth factor receptors discussed in this section, many others such as FGFR, PDGFR, IGFR and VEGFR, are known to be important in anoikis resistance in a range of other cancers (Hilmi et al., 2008; Molhoek et al., 2011; Paoli et al., 2013; Chen et al., 2016), and likely also have a role in melanoma. However, little evidence is currently available, and they are therefore outside the scope of this review.

#### 2.2.1 EGFR

A switch in expression levels of epidermal growth factor receptors is demonstrated to occur during melanoma progression and correlates with the acquisition of invasive or proliferative cell behaviour (Tsoi et al., 2018). EGFR (*ERBB1*) is a neural crest-associated gene that is found to be highly expressed in subpopulations of melanoma tumour cells exhibiting an undifferentiated or neural crest stem cell-like (NCSC) gene expression signature, including upregulation of *SOX9*, *NGFR* and *AXL*, downregulation of *SOX10,* and a highly invasive phenotype (Sun et al., 2014; Tsoi et al., 2018). EGFR has been identified as a driver of resistance to therapeutic MAPK pathway inhibition (Abel et al., 2013; Dugo et al., 2015), where autocrine activation of EGFR stimulates activation of the downstream MAPK and PI3K-Akt signalling pathways (Oberst et al., 2008; Sun et al., 2014; Liu S. et al., 2021). Conversely, subpopulations of cells with a proliferative phenotype preferentially express *ERBB3* (Tsoi et al., 2018); with *ERBB1* and *ERBB3* expression shown to be mutually exclusive in melanoma cell lines (Dugo et al., 2015). However, the ERBB3 molecule demonstrates no kinase activity except when dimerised with EGFR or ERBB2, while ERBB2 expression is reported to be very low or absent in melanoma (Liu S. et al., 2021, Ueno et al., 2008; Inman et al., 2003). This suggests that a switch in EGF receptor expression occurs, with ERBB3 slowly downregulated and EGFR upregulated during the transition to an invasive phenotype in order to drive MAPK and PI3K-Akt pathway activity and ultimately metastasis. However, these findings contradict a study utilising B16-BL6 melanoma cells indicating that ERBB3, when dimerised with EGFR, is essential for tumour metastasis both *in vitro* and *in vivo*, and upregulates mesenchymal genes downstream of MAPK, JNK and PI3K-Akt pathway activation (Ueno et al., 2008). Nevertheless, the study by Ueno and others is based on a single murine melanoma cell line which likely fails to recapitulate the intratumoural heterogeneity found in a patient tumour, and thus the switch observed.

In conjunction with overexpression of EGFR, metastatic melanoma cells are demonstrated to overproduce the epidermal growth factor (EGF) ligand. EGF can subsequently act in an autocrine signalling manner allowing melanoma cells to produce their own growth and survival signals, as well as in a paracrine manner acting on endothelial cells to drive neoangiogenesis (Bracher et al., 2013). The hyperactivated EGF signalling cascade promotes expression of MMPs, adhesion molecules and initiators of EMT, subsequently driving invasion and metastasis (Kajanne et al., 2007; Lafky et al., 2008; Hardy et al., 2010; Kim et al., 2011; Zuo et al., 2011).

Overexpression and hyperactivation/hyper-phosphorylation of EGFR has been linked to anoikis resistance in a range of cancers, including hepatocellular carcinoma (Lim et al., 2020), GBME (Talukdar et al., 2018), breast (Oberst et al., 2008), lung (Chunhacha et al., 2013) and prostate cancer (Giannoni et al., 2009). In epithelial cells, hyperactivation of EGFR results in abrogated activity of pro-apoptotic Bim through the maintenance of MAPK pathway signalling, allowing the survival of cells upon ECM detachment (Reginato et al., 2003). While investigating the effects of reduced pH on the survival and metastasis of human melanoma cells, Peppicelli and others identified that cells possessing an anoikis resistant phenotype expressed high levels of EGFR, and reduced levels of cleaved PARP-1 (Peppicelli et al., 2019). These cells additionally displayed enhanced motility and invasion through matrigel, and expressed markers of a mesenchymal-like phenotype including N-cadherin. Furthermore, CTCs from melanoma are demonstrated to express receptors from the EGFR family (Tsao et al., 2018). While the role for EGFR signalling in anoikis resistance in melanoma cells remains poorly defined, it is hypothesised that the sustained survival signalling provided by autocrine EGF/EGFR activation within clusters of melanoma cells contributes to the survival of disseminated cells.

**FIGURE 4:**
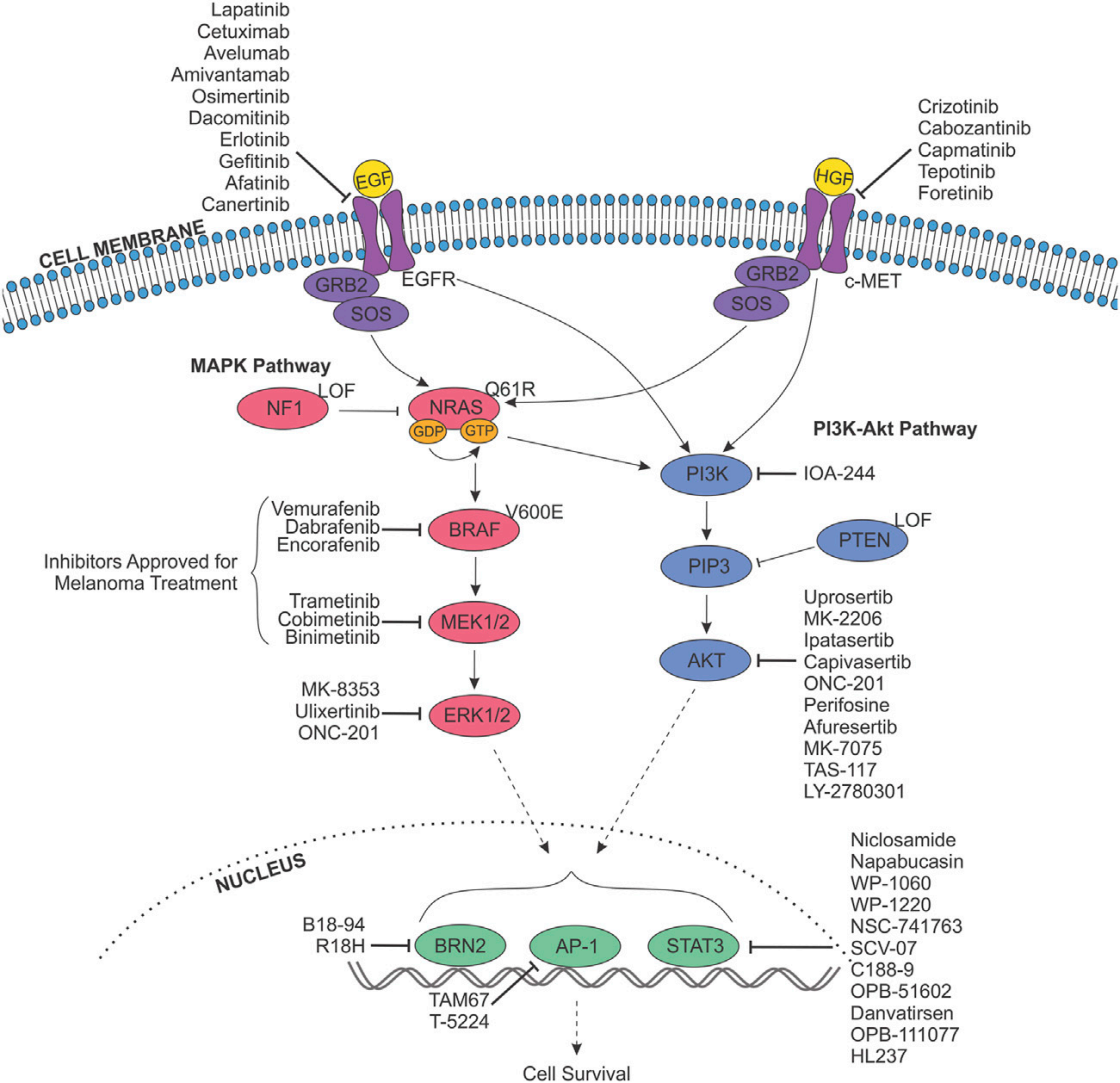
Targeting receptor tyrosine kinase signalling pathways in melanoma anoikis resistance. The MAPK (pink) and PI3K-Akt (purple) signalling pathways are constitutively activated in melanoma and stimulated by the binding of a growth factor (Epidermal Growth Factor (EGF)/Hepatocyte Growth Factor (HGF); yellow) to their respective receptor tyrosine kinase at the cell membrane (EGFR/c-MET). Oncogenic activation of these pathways is due to one of three proteins, whose genes are mutated with high frequently in melanoma; BRAF, NRAS and NF1. Most commonly, the BRAF gene harbours a mutation resulting in the V600E alteration, while NRAS usually possesses mutations leading to the Q61R variant, and NF1 is frequently effected by a genetic alteration resulting in loss of function (LOF). These variations result in abnormal signalling to downstream effectors, resulting in altered proliferation, differentiation, apoptosis, cell survival and metabolism; inevitably driving melanoma initiation and progression. As such, components of the MAPK pathway are targeted with small molecule inhibitors for the therapeutic treatment of melanoma, and have been approved as either single-agent therapies or in combination. Inhibitors approved for treatment of melanoma are indicated. In addition, inhibitors targeting EGFR, c-MET, the PI3K-Akt pathway, and downstream transcription factors (green) exist, and have the potential to block anoikis resistance. Indicated inhibitors have been sourced from data from all cancers.

A range of small molecule inhibitors and monoclonal antibody therapies have been designed to target EGFR hyperactivation in cancers of epithelial origin (Figure 4). Lapatinib is an antineoplastic small molecule kinase inhibitor registered by the FDA (2007) for the treatment of patients with advanced metastatic breast cancer (HER2/ERBB2+) in conjunction with the chemotherapeutic agent capecitabine (Ryan et al., 2008). Lapatinib prevents phosphorylation of multiple RTKs, including EGFR, ERBB2, ERK1/2 and Akt and has demonstrated efficacy in clinical trials covering other malignancies (NCT00486954, NCT00949455, NCT02230553, NCT00095940, NCT01184482, NCT04608409). Similarly, three monoclonal antibody therapies targeting EGFR have been registered by the FDA. Cetuximab (2004) was first approved for the treatment of metastatic colorectal carcinoma (CRC) and head and neck squamous cell carcinoma (HNSCC), while avelumab (2017, NCT03089658) and amivantamab (2021, NCT04599712) were approved for the treatment of metastatic Merkel cell carcinoma and EGFR-mutant NSCLC, respectively. A multitude of other ERBB inhibitors (ERBBi) have demonstrated efficacy in clinical trials, with the most notable examined in phase IV trials: osimertinib (NCT03853551), dacomitinib (NCT04511533), erlotinib (NCT01230710, NCT00949910, NCT01402089, NCT01609543, NCT01066884), gefitinib (NCT00770588, NCT01203917, NCT00608868, NCT01000740), afatinib (NCT02514174, NCT02208843).

Extensive preclinical studies have examined the effects of ERBBi in metastatic melanoma. A study by Girotti and others demonstrated that BRAFi-resistant melanoma cell lines express high levels of phosphorylated EGFR, and secrete increased levels of EGF (Girotti et al., 2013). Treatment of resistant A375 cells with gefitinib reduced *in vitro* proliferation and invasion. Subsequent *in vivo* treatment of BRAFi-resistant A375 tumour xenografts with combination gefitinib plus vemurafenib (PLX4720) synergistically reduced tumour volume, compared to vemurafenib or gefitinib alone. As BRAFi treatment has been demonstrated to promote SOX10-low/EGFR-high expressing cell populations, an independent study investigated the same inhibitor combination in the A375 cell line in the context of SOX10 knockout (Sun et al., 2014). However, the combination of gefitinib and vemurafenib did not lead to proliferation arrest in this setting, suggesting that further investigation is required to identify the specific cohort of patients that may benefit from ERBB inhibition. Interestingly, a study comparing single-agent treatment using gefitinib, erlotinib or lapatinib *versus* the pan-ERBB inhibitor (targeting EGFR, ERBB2,3 and 4), canertinib (CI-1033), revealed increased ability of the multi-kinase inhibitor to reduce *in vitro* proliferation of both BRAF-wild-type (WT) and BRAF^V600E^ melanoma cell lines (Ng et al., 2014). Canertinib was additionally shown to synergise with vemurafenib *in vitro,* significantly reducing the IC_50_ of vemurafenib in 4 of 6 melanoma lines assessed.

ERBB4 is found to be somatically mutated in 19% of melanomas, with the majority of alterations occurring in the extracellular domain of the receptor, resulting in enhanced kinase activity (Prickett et al., 2009). Importantly, ERBB4 mutations increased the ability of melanoma cells to grow under anchorage-independent conditions, as assessed by the ability of SK-MEL-2 cells to form colonies in soft agar. Treatment with lapatinib was demonstrated to significantly reduce cell growth in ERBB4-mutant melanoma cells lines at 10- to 250- fold higher sensitivity (IC_50_) than ERBB4 WT cells, resulting in dose-dependent inhibition of ERBB4 auto-phosphorylation and downstream Akt signalling. However, a phase II clinical trial evaluating the safety and efficacy of lapatinib in treatment refractory stage IV melanoma patients carrying ERBB4 mutations (NCT01264081) was terminated with 30/34 patients failing to complete the trial. Nevertheless, the *in vitro* and *in vivo* evidence supporting the use of ERBBi in melanoma is strong, and further investigation into their use in melanoma patients with high levels of EGFR expression is warranted, particularly given the evidence for its role in driving anoikis resistance.

#### 2.2.2 c-MET/HGF

c-MET (encoded by the proto-oncogene, *MET*) is a transmembrane RTK activated by its’ ligand, hepatocyte growth factor (HGF). The c-MET/HGF signalling pathway normally acts in a paracrine manner, with HGF secreted by mesenchymal cells and acting on surrounding epithelial cells (Li et al., 2001b). The conformation change induced by HGF binding, and the subsequent auto-phosphorylation of the homodimerised receptor subunits at Y1234 and Y1235, results in the recruitment of downstream effector proteins such as STAT3 and Grb2 (Organ and Tsao, 2011). Guanine nucleotide exchange factors (GEFs), including SOS, are subsequently recruited resulting in the stimulation of the MAPK pathway via the activation of Ras-GDP to Ras-GTP, as well as the initiation of PI3K-Akt pathway signalling (Zhang et al., 2018). During embryonic development, c-MET/HGF signalling plays a crucial role in the survival and migration of myogenic progenitor cells through stimulation of EMT (Bladt et al., 1995; Jeon and Lee, 2017).

Expression of c-MET is detected in both keratinocytes and melanocytes in the dermis and epidermis of the skin (Saitoh et al., 1994). However, following oncogenic transformation, melanoma cells gain expression of HGF, allowing autocrine c-MET/HGF signalling that functionally decouples melanoma cells from keratinocytes, facilitating invasion and migration (Natali et al., 1993; Otsuka et al., 1998; Rusciano et al., 1998; Li et al., 2001b). Studies subsequently established that melanoma cells with high c-MET/HGF autocrine signalling have an increased propensity for metastasis to the liver (Lin et al., 1998; Otsuka et al., 1998). Expression of c-MET in BRAF^V600E^ melanoma cell lines, mouse xenografts and patient tumours is further demonstrated to contribute to vemurafenib resistance under hypoxic conditions (Qin et al., 2016). At a cellular level, c-MET is localised to sites of cellular adhesion (Kenessey et al., 2010), and co-immunoprecipitates with adhesion molecules such as E-cadherin and desmoglein 1 (Li et al., 2001b). A study by Koefinger and others demonstrated that expression of HGF in melanoma cells induces a switch in cadherin expression from E-to N-cadherin, driven by the downregulation of Slug and upregulation of Twist (Koefinger et al., 2011). Overexpression of c-MET also promotes proteolytic processes associated with invasion through the upregulation of urokinase-type plasminogen activator (uPA) and MMP expression (Rusciano et al., 1998; Tanaka et al., 2021).

Given the role of c-MET in melanoma invasion and migration, and its association with adhesion molecules and EMT processes, the involvement of c-MET/HGF signalling in anoikis resistance is unsurprising. An early study in hepatocytes revealed that c-MET functionally sequesters the Fas death receptor to prevent apoptosis, suggesting that a high c-MET to Fas ratio may be involved in cancer cell survival (Wang et al., 2002). In accordance with this hypothesis, c-MET is demonstrated to contribute to anoikis resistance in detached ovarian cancer cells through activation of MAPK and PI3K pathways (Tang et al., 2010), as well as in gastric cancer (Toiyama et al., 2012), HNSCC (Zeng et al., 2002) and prostate cancer (Dai and Siemann, 2012). In a study by Pierce and others demonstrating increased anoikis resistance in melanoma cells following overexpression of the BRN2 transcription factor, a significant increase in c-MET expression and phosphorylation were observed (Pierce et al., 2020). Comparable with autocrine EGFR signalling discussed previously, autocrine c-MET/HGF signalling in disseminated cells and CTC clusters is hypothesised to contribute to the survival signalling necessary to evade anoikis during melanoma metastasis.

Due to the high prevalence of c-MET hyperactivation in a variety of cancers, inhibitors have been designed to target the molecule for therapeutic purposes (Figure 4). Crizotinib is an ATP competitive inhibitor against c-MET, as well as ALK, FDA registered (2011) for the treatment of patients with advanced or metastatic NSCLC. Preclinical evidence in uveal melanoma revealed the feasibility of crizotinib treatment, demonstrating reduced *in vitro* viability and migration, and a significant reduction in *vivo* formation of macro-metastases (Surriga et al., 2013). Furthermore, multiple studies have investigated combining crizotinib with other inhibitors for the treatment of cutaneous melanoma. The combination of crizotinib with the ERBBi afatinib demonstrated synergistic cytotoxic effects, significantly reducing 2D and 3D invasion, migration and colony formation independent of BRAF/NRAS mutation status, and resulting in decreased tumour volume *in vivo* (Das et al., 2019; Das et al., 2020). Importantly, a phase I study investigating the use of crizotinib in combination with vemurafenib in advanced melanoma patients has demonstrated safety and efficacy [NCT01531361 (Janku et al., 2021)]. Single-agent treatment with crizotinib is under investigation in a phase II trial for the treatment of uveal melanoma patients (NCT02223819), while a phase I/II study is currently recruiting patients with solid tumours carrying *GNAQ/11* mutations or *PRKC* gene (encoding PKC protein family members) fusions (NCT03947385), as these alterations are shown to result in HGF hypersecretion and hyperactivation of c-MET (Kermorgant et al., 2004; Surriga et al., 2013).

Cabozantinib is a small molecule inhibitor targeting a range of RTKs including c-MET, KIT, VEGFR-1/2/3, AXL and TRKB. It was first registered by the FDA (2012) for the treatment of patients with progressive and unresectable advanced medullary thyroid cancer (MTC), and has subsequently received FDA registration for use in treatment-refractory advanced renal cell carcinoma (2016), hepatocellular carcinoma (HCC) (2019), and as a first line treatment for differentiated thyroid cancer (DTC) (2021) and RCC (2021). A preclinical study investigating cabozantinib treatment of cell lines derived from melanoma brain metastases demonstrated the ability of the inhibitor to significantly reduce viability of cells in monolayer and 3D spheroid cultures, reduce migration and colony formation, and downregulate phosphorylation of Akt and MEK1/2 (Mannsåker et al., 2021). Phase I and II trials investigating the response of uveal and cutaneous melanoma patients to cabozantinib treatment have been completed [NCT01709435, NCT00940225 (Daud et al., 2017), NCT01835145 (Luke et al., 2020)].

Capmatinib is a c-MET inhibitor registered by the FDA (2020) for the treatment of metastatic NSCLC patients with *MET* exon 14 skipping mutations. Preclinical studies have evaluated the treatment of melanoma patient-derived xenografts (PDXs) with single-agent capmatinib, and in combination with encorafenib (BRAFi), binimetinib (MEKi) or both (Krepler et al., 2016). The study revealed complete tumour regression in 100% of mice treated with capmatinib + binimetinib + encorafenib, with no evidence of therapy resistance. A phase II clinical trial is currently recruiting advanced melanoma patients for treatment with the triple combination (NCT02159066). Similarly, tepotinib was FDA registered in 2021 for the treatment of NSCLC, and demonstrated evidence against melanoma in preclinical studies. Treatment of WM451 cells suppressed invasion and migration, and induced apoptosis by blocking PI3K-Akt signalling resulting in reduced Bcl-2, increased Bax expression and caspase-3 cleavage, accompanied by a switch in N- to E-cadherin expression suggesting suppression of EMT (Jing et al., 2022).

Foretinib (EXEL-2880, GSK1363089), a c-Met and VEGFR2 inhibitor, has demonstrated a high level of efficacy in clinical trials for other cancer types (NCT00742131, NCT00742261, NCT00743067), and in preclinical studies. Treatment of B16-F10 melanoma cells with foretinib blocked anchorage-independent growth under hypoxic conditions *in vitro*, and significantly reduced spontaneous metastasis to the lungs of mice in a dose-dependent manner (Qian et al., 2009). Similar to crizotinib, combining foretinib treatment with ERBBi gefitinib or lapatinib demonstrated synergistic cytotoxic effects on melanoma cells (Dratkiewicz et al., 2018; Simiczyjew et al., 2019), while combination treatment with vemurafenib produced comparable effects (Dratkiewicz et al., 2019).

In addition to the direct effect of c-MET inhibitors on tumour growth and invasion, treatment with c-MET inhibitors foretinib, crizotinib and cabozantinib are demonstrated to reduce the viability of vascular endothelial cells immortalised from melanoma tumours grown in immunocompetent mice (Jenkins et al., 2021), revealing the potential efficacy of c-METi on tumour cells, stromal cells and CTCs.

### 2.3 Signalling pathways

#### 2.3.1 FAK signalling

First linked to anoikis resistance in mammary epithelial cells by Frisch et al. (1996), focal adhesion kinase (FAK/*PTK2*) plays a role in tyrosine kinase signalling where it interacts with the β subunit of integrins in the cytosol [reviewed in (Paoli et al., 2013)]. Cell adhesion to ECM components through integrin binding results in rapid activating phosphorylation of FAK (p-FAK) at tyrosine residues 397 and 576, resulting in the recruitment of proteins including Src kinase, the actin cytoskeleton-binding paxillin and adaptor proteins such as Grb2, activating multiple downstream pathways including MAPK and PI3k-Akt signalling (Goundiam et al., 2012). FAK also has a distinct role independent of focal adhesions as a result of a nuclear localisation signal within the FERM domain of the protein (Aplin et al., 1998; Parsons et al., 2000). Normally, interrupting focal adhesions through cellular detachment results in rapid dephosphorylation of FAK and its cleavage facilitated by caspase-3, -6 and -9, resulting in translocation to the nucleus where FAK prevents activation of wild-type p53 signalling, triggering intrinsic apoptosis (Del Mistro et al., 2022).

In melanoma, studies indicate that inappropriate phosphorylation of FAK upon cell detachment contributes to anoikis resistance by allowing persistent survival signalling (Hess et al., 2005). Studies utilising B16-F10 murine melanoma cells cultured on poly-HEMA (an ultra-low attachment coating) demonstrate increased expression of p-FAK, alongside increased expression of downstream molecule RhoA, and increased activating phosphorylation of Akt (p-Akt) and ERK1/2 (p-ERK1/2) when compared to the Swiss 3T3 anoikis sensitive cell line used as a control (Goundiam et al., 2010). Interestingly, a study demonstrated that stabilisation of p-FAK through interaction with the cell cycle regulator p14^ARF^ and subsequent sumoylation contributes to anoikis resistance (Vivo et al., 2017). Mutant forms of p14^ARF^ containing point mutations identified in melanoma were able to stabilise p-FAK more effectively than wild-type p14^ARF^. Comparably, FAK knockdown (siFAK) in B16-F10 cells was shown to suppress migration and metastasis *in vivo*, with decreased number of metastatic nodules present in the lungs of mice upon siFAK in comparison to controls (Pei et al., 2017). FAK knockdown decreased expression of p-ERK1/2, p-STAT3 and increase expression of E-cadherin, while siRNA against Akt and PI3K reduced p-FAK expression (Goundiam et al., 2012; Pei et al., 2017).

Small molecule inhibitors that target FAK activity have been explored in both phase I and II trials for use in a range of advanced solid and haematological malignancies, and may represent a viable option for repurposing to target melanoma CTCs. Defactinib (VS-6063, PF-04554878) is a specific inhibitor of FAK with demonstrated antioangiogenic and antineoplastic activities. Defactinib is shown to be safe in healthy subjects, and has demonstrated efficacy against advanced malignancies in phase I trials (NCT02913716, NCT02546531 and NCT01943292). Interestingly, in a study utilising melanoma cells from patients who relapsed following treatment with BRAF or MEK targeted inhibitors, treatment with the FAK inhibitor (FAKi) defactinib was able to resensitise cells to killing by MAPK pathway inhibition (Del Mistro et al., 2022). Other FAKi yet to be examined in melanoma are summarised in Table 2.

The literature suggests that clusters of tumour cells in circulation have greater metastatic potential and increased ability to evade anoikis than single tumour cells (Hou et al., 2011; Hou et al., 2012). A study revealed the potential efficacy of using FAKi to target anoikis resistant tumour cells in circulation (Au et al., 2016). It was demonstrated that CTC clusters are able to transit through capillaries with over 90% efficacy by unfolding into single-file chains of cells. Use of a FAK inhibitor tool compound (FAK I-14) resulted in a significantly greater likelihood of CTC clusters being disrupted when transiting through capillaries than untreated clusters (Figure 1).

#### 2.3.2 MAPK signalling pathway

Melanomagenesis occurs primarily through the acquisition of pathogenic mutations in genes that encode key components of the Mitogen-Activated Protein Kinase (MAPK) signalling pathway (Guo et al., 2020). Mutations in *BRAF,* (*N/H/K*) *RAS* or *NF-1* were detected in 52%, 30.9% and 14% of patients in the Cancer Genome Atlas Network (TCGA) dataset, respectively (Akbani et al., 2015). These mutations cause constitutive activation of the MAPK pathway, driving processes such as proliferation, differentiation and abrogated apoptosis via the activation of a kinase cascade resulting in downstream nuclear transcription factor phosphorylation and activation (Guo et al., 2020). MAPK signalling is demonstrated to be stimulated by microenvironmental growth factors, and result in activation of the EMT-TFs (Lamouille et al., 2014). The downregulation of Slug and Zeb2, and upregulation of Zeb1, Snail and Twist1 promote differentiation and metastasis in melanoma cells (Hoek et al., 2004; Kuphal et al., 2005; Caramel et al., 2013). However, the role of Slug in melanoma appears to be highly context dependent, with other studies demonstrating increased expression in invasive cell populations (Fenouille et al., 2012; Pearlman et al., 2017).

Accordingly, multiple small molecule inhibitors against key components of this pathway have been registered by the FDA for use in metastatic melanoma (Figure 4). Vemurafenib (approved in 2011) and dabrafenib (2013) target and inhibit mutant-BRAF (BRAFi) (Jenkins and Fisher, 2021). In addition, between 2014 and 2018, three regimens that combine the use of a BRAFi with an inhibitor against MEK (MEKi) were approved for use. The combinations of dabrafenib + trametinib, vemurafenib + cobimetinib, and encorafenib + binimetinib have demonstrated efficacy against melanomas with mutations in both the *NRAS* and *BRAF* genes, and are shown to improve therapy response and overall PFS (Flaherty et al., 2012; Ascierto et al., 2013; Larkin et al., 2014; Long et al., 2015; Robert et al., 2015). However, following treatment with such inhibitors, multiple distinct and drug resistant subpopulations of melanoma cells persist, and quickly drive relapse in a proportion of patients (Fedorenko et al., 2011; Rambow et al., 2018; Rambow et al., 2019).

Together with its role in driving melanoma formation and progression the MAPK signalling pathway contributes significantly to the acquisition of resistance to cell death by anoikis in melanoma cells. Unlike primary human epidermal melanocytes, melanoma cell lines carrying the BRAF^V600E^ alteration demonstrate enhanced cellular survival and reduced apoptosis when plated on agar, as measured by caspase-3 cleavage (Boisvert-Adamo and Aplin, 2006). It was identified that mutant-BRAF drives anoikis resistance through constitutive activation of MAPK signalling, resulting in the depletion of two pro-apoptotic Bcl-2 family members; the Bcl-xl/Bcl-2-associated death promoter (Bad) and Bcl-2-interacting mediator of cell death (Bim), while simultaneously increasing expression of anti-apoptotic myeloid cell leukemia-1 (Mcl-1) (Boisvert-Adamo and Aplin, 2008; Boisvert-Adamo et al., 2009; Goldstein et al., 2009). Consistent with these findings, siRNA-mediated ablation of BRAF, or pharmacological inhibition of MEK, were demonstrated to induce susceptibility to anoikis in melanoma cells (Boisvert-Adamo and Aplin, 2006). However, it is clear from the vast number of studies detailing intrinsic and acquired resistance to single-agent and combination MAPKi therapy (Fedorenko et al., 2011; Winder and Virós, 2018), that the use of BRAF or MEK targeted inhibitors alone is insufficient to block anoikis resistance long-term in patients.

Reactivation of ERK1/2 is considered a primary mechanism of MAPKi therapy resistance (Lee et al., 2020). Given its role in cell cycle progression and evasion of apoptosis, inhibitors against ERK1/2 are being investigated in a range of advanced and metastatic malignancies (Figure 4). MK-8353 is a selective inhibitor that targets both phosphorylated and unphosphorylated ERK1/2. Preclinical evaluation in an *in vivo* human xenograft model derived from the BRAF^V600E^-mutant melanoma cell line SK-MEL-28, demonstrated significant reduction in tumour volume compared to untreated controls (Moschos et al., 2018). Despite reporting efficacy and safety in a phase I trial of patients with advanced solid tumours (NCT01358331), MK-8353 demonstrated an anti-tumour response in only 3 of 8 BRAF^V600E^ melanoma patients, with all patients withdrawing from the trial as a result of disease progression or adverse effects. MK-8353 has also undergone testing in a phase 1b trial in combination with the ATP competitive MEK1/2 inhibitor Selumetinib in advanced or metastatic solid tumours in an attempt to suppress MAPK therapy resistance (NCT03745989), and is likewise currently under investigation in combination with the immunotherapy pembrolizumab (NCT02972034). Similarly, Ulixertinib (BVD-523) in an ATP-competitive inhibitor against ERK1/2 that has successfully completed phase I/II trials examining dosage and safety, displaying favourable pharmacokinetics in NRAS-mutant and BRAF^V600E^ advanced solid malignancies, as well as those carrying BRAF mutations at non-V600 sites (NCT01781429) (Sullivan et al., 2018; Wu J. et al., 2021). Interestingly, ulixertinib was recently used in a phase II trial for the treatment of patients with metastatic uveal melanoma (NCT03417739). However, the therapy failed to demonstrate anti-tumour activity, with those treated demonstrating a median overall survival of 6.9 months (3.2–8.3), and 38.46% of participants reporting serious adverse events as a result of the treatment (Buchbinder et al., 2020). In addition to the use of ERKi, the current gold-standard treatment combining the use of targeted inhibitors with immunotherapies suggests there may be efficacy in further combining BRAF or MEK inhibitors with those against other known mediators of anoikis resistance described in this review to target CTCs, especially given the considerable level of crosstalk between MAPK, PI3K-Akt, EGFR, FAK and other key signalling pathways in melanoma.

#### 2.3.3 PI3K-Akt signalling

The Phosphatidylinositol 3-Kinase Akt (PI3K-Akt) signalling pathway has been demonstrated to synergise with oncogenic MAPK signalling to increase proliferation and disease progression (Atefi et al., 2011; Greger et al., 2012). PI3K-Akt signalling is constitutively activated by mutant *NRAS* (Johnson and Puzanov, 2015), as well as the *PTEN* tumour suppressor which is found to be effected by loss of function mutations in approximately 20% of *BRAF*-mutant melanomas, resulting in Akt activation (Shull et al., 2012; Akbani et al., 2015). Overall, expression of Akt is elevated in approximately 70% of malignant melanomas resulting in the dysregulation of downstream effector molecules, such as mTOR (Pearlman et al., 2017). Studies propose that melanoma invasiveness may be regulated by a PI3K-PAX3-BRN2 axis, with inhibition of PI3K signalling shown to reduce invasion and downregulate expression of both PAX3 and BRN2 (Bonvin et al., 2012). In epithelial cells, activation of PI3K-Akt signalling drives the process of EMT through the activation of the EMT-TFs, protecting against death by anoikis in suspension culture conditions (Khwaja et al., 1997; Xu et al., 2015). Mechanistically, Akt activity is able to abrogate apoptosis via the phosphorylation of Bim and Bad pro-apoptotic proteins, inhibiting caspase-9 activity and transcription of the Fas death receptor ligand (Qi et al., 2006; del Peso et al., 1997; Brunet et al., 1999; Kennedy et al., 1999).

In melanoma, PI3K-Akt signalling is thought to contribute to anoikis resistance. Early research suggested the pathway may act as a secondary survival cue, protecting cells against apoptosis in the absence of mutant-*BRAF* (Boisvert-Adamo and Aplin, 2006). In the presence of mutant-*BRAF, PTEN-*deficient melanoma cells express constitutively active Akt3, which protects against apoptosis by upregulating Bim and Bmf (Shao and Aplin, 2010). When cultured on poly-HEMA, B16-F10 cells demonstrated a transient increase in Akt phosphorylation, while total protein level was unchanged (Goundiam et al., 2010). A subsequent study revealed that inhibition of Akt activation, as well as downstream RhoA and RhoC expression, resulted in induction of anoikis through the inactivation of the FAK signalling pathway (Goundiam et al., 2012). Similarly, two consecutive studies by Toricelli et al. validated these findings, revealing that inhibition of PI3K or Akt proteins reversed anoikis resistance in melanoma cells (Toricelli et al., 2013; Toricelli et al., 2017). Inhibition of Timp1, an MMP inhibitor whose overexpression activates PI3K-Akt signalling, induced sensitivity to anoikis *in vitro* and reduced tumour volume and metastatic colony formation *in vivo.* Subsequent studies have shown that anoikis resistance induced via the knockdown of α2, α3β1 or α5β1 integrins can be rescued via the specific inhibition of Akt (Kozlova et al., 2019; Kozlova et al., 2020). Furthermore, MIST1 and SNAI1 transcription factors are thought to contribute to anoikis resistance by directly repressing *PTEN* (Lee et al., 2018), while knockdown of the NCAM adhesion molecule induced apoptosis in melanoma cells by suppressing Akt activation (Li et al., 2020).

Given the frequency of Akt hyperactivation in melanoma, a range of inhibitors have been designed against its’ isoforms and may be repurposed in order to target anoikis resistance (Table 2; Figure 4). Uprosertib is an orally bioavailable inhibitor against Akt. Preclinical evidence demonstrated the potential efficacy of combining uprosertib with MEKi against a range of cancer cell lines with mutations in the *BRAF* or *KRAS* genes (NCT01935973, NCT01989598) (Dumble et al., 2014). However, a phase II study investigating the combination in *BRAF/NRAS* wild-type, and *NRAS-*mutant melanoma patients (NCT01941927) revealed no improvement to overall or progression-free survival (Algazi et al., 2018). A similar phase II trial in stage IV uveal melanoma patients (NCT01979523) revealed analogous results, with dose reductions required due to the frequency of adverse events (Shoushtari et al., 2016), as predicted in the phase I dose-escalation study [NCT01138085 (Tolcher et al., 2020)]. Despite this, a phase I/II trial into uprosertib plus dabrafenib and trametinib in stage III/IV metastatic solid cancers, including melanoma, is currently underway (NCT01902173).

MK-2206 inhibits Akt, blocking downstream PI3K-Akt signalling. Its’ anti-tumour activity has been assessed in clinical trials against a range of cancers (NCT01071018, NCT00670488, NCT00848718, NCT01283035, NCT01604772, NCT01349933, NCT01802320, NCT01333475, NCT01258998, NCT01481129, NCT01253447, NCT01231919, NCT01369849, NCT01277757) (Molife et al., 2014). Preclinical studies treating melanoma cells *in vitro* with MK-2206 resulted in a concentration-dependent downregulation of phosphorylated Akt, inhibition of cell growth and colony formation, and induced apoptosis through altered expression of Bax, and increased ROS generation (Quast et al., 2013; Petit et al., 2019). MK-2206 has further demonstrated efficacy in combination with binimetinib, where treatment resulted in a synergistic reduction in tumour volume (Petit et al., 2019), as well as in combination with the mTOR inhibitor everolimus (Ciołczyk-Wierzbicka et al., 2020), and vemurafenib (Su et al., 2012; Thang et al., 2015). Interestingly, a phase I trial investigating MK-2206 in combination with paclitaxel and carboplatin for the treatment of two patients with *BRAF-*wild-type stage IV melanoma reported long-term, enhanced responses to chemotherapy [NCT00848718 (Rebecca et al., 2014)]. However, a phase II clinical trial investigating MK-2206 plus selumetinib (MEK1/2 inhibitor) in stage III/IV melanoma patients who previously failed vemurafenib or dabrafenib treatment was terminated due to slow accrual (NCT01519427).

The small molecule Akt inhibitor Ipatasertib (GDC-0068) has been examined in phase I trials against breast, ovarian and prostate cancers (NCT03840200, NCT01562275, NCT01362374) (Yan et al., 2013), as well as in phase II trials for gastric cancers (NCT01896531) and in combination with paclitaxel for breast cancer treatment (NCT02301988, NCT02162719). While its efficacy in melanoma patients is yet to be examined, ipatasertib is demonstrated to prevent growth of the PTEN-null melanoma tumours *in vivo* (Saura et al., 2017). Similarly, capivasertib (AZD-5363) targets all isoforms of Akt, and has completed phase I trials against solid tumours (NCT04742036), haematological malignancies (NCT04944771) and prostate cancer (NCT04087174). In a study by Dinavahi and others, simultaneously administering capivasertib with the WEE1 inhibitor AZD-1775, synergistically reduced melanoma cell survival *in vitro* and tumour growth *in vivo* by driving increased expression of p53 and blocking Akt-mediated activation of FOXM1 (Dinavahi et al., 2018).

ONC-201 (TIC-10) is an orally available small molecule inhibitor with activity against both Akt and ERK. The efficacy of ONC-201 for the treatment of advanced malignancies has been investigated in phase I and II (NCT02324621, NCT02250781, NCT02609230, NCT03394027). Interestingly, as ONC-201 is water-soluble and able to cross the blood-brain barrier, phase II/III clinical trials are currently recruiting participants to examine its efficacy against gliomas (NCT05476939, NCT05009992). While yet to be trialled in melanoma patients, pre-clinical evidence demonstrated treatment of melanoma cell lines with ONC-201 reduced colony formation and migration *in vitro,* decreased expression of p-Akt and p-ERK, and resulted in a significant reduction in tumour volume (Wagner et al., 2017). Further, combination treatment with bortezomib, an inhibitor of the ubiquitin-proteasome pathway, demonstrated a synergistic ability to reduce cell viability and induce apoptosis (Takács et al., 2021). Although ONC-201 has been examined in clinical trials as a specific inhibitor against Akt and ERK, conjecture surrounding its mechanism of action remains, with studies suggesting it may indirectly modulate Akt activity as a downstream effect of dopamine receptor D2 inhibition (McSweeney et al., 2020; Prabhu et al., 2020).

Perifosine (KRX-0401) is a small molecule inhibitor against Akt that has been examined in phase II trials against a range of cancers (NCT00389077, NCT00873457, NCT00455559, NCT00590954, NCT00448721, NCT00498966, NCT00375791, NCT00398879, NCT00053924, NCT00059982). Despite evidence that perifosine treatment of melanoma cells reduces expression of p-Akt (Liu and Xing, 2012), a phase II trial in metastatic cutaneous melanoma patients failed to demonstrate efficacy [NCT00053781 (Ernst et al., 2005)]. Afuresertib (GSK-2110183) is an orally available small molecule inhibitor of Akt. A phase I/II dose escalation study combining afuresertib with trametinib in patients with advanced solid tumours observed a partial response in a patient with BRAF-wild-type melanoma, however the combination therapy was poorly tolerated with extensive adverse effects recorded [NCT01476137 (Tolcher et al., 2015)].

Furthermore, there has been growing investigation into the use of inhibitors targeting overexpression of PI3K directly in breast cancer and haematological malignancies [reviewed in (Vanhaesebroeck et al., 2021)], which is beginning to expand into other solid tumour types with a phase I trial examining the safety and tolerability of IOA-244 in uveal melanoma patients currently recruiting participants (NCT04328844). In addition, given that constitutive activation of the PI3K-Akt pathway activates downstream mTOR signalling to drive survival, motility, invasion and proliferation, multiple studies have investigated the use of mTOR inhibitors in combination with other therapies for the treatment of metastatic melanoma patients [recently reviewed in (Chamcheu et al., 2019)]. Such combination treatments may also be efficacious in the targeting of anoikis resistant melanoma cells.

### 2.4 Transcription factors

#### 2.4.1 BRN2

BRN2, the POU domain transcription factor encoded by the gene *POU3F2*, has been linked to melanoma progression in the phenotype switching model as a potential driver of invasive behaviour (Cook and Sturm, 2008; Hoek and Goding, 2010) [reviewed in (Fane et al., 2019)]. BRN2 plays a role in the delineation of neural crest cells to the melanocytic lineage during embryonic development, similar to other factors such as SOX10 and the Microphthalmia-associated transcription factor (MITF) (Thomson et al., 1995; Cook et al., 2003). BRN2 is expressed in melanoma tissues (Sturm et al., 1991; Thomson et al., 1993), with 10-fold higher expression observed in melanoma cell lines in comparison to normal melanocytes (Eisen et al., 1995). Early research revealed that inhibition of BRN2 expression results in complete loss of tumour formation in mice, and the loss of melanocyte markers including MITF (Thomson et al., 1995). Subsequently, it was identified that BRN2 expression is inversely correlated with, and mutually exclusive to MITF expression in patient tumours and xenografts (Goodall et al., 2008). MITF and BRN2 therefore regulate opposing functions in the phenotype switching phenomenon by virtue of their ability to modulate each other; BRN2 represses expression of MITF by directly binding to its promoter (Goodall et al., 2008) as well as indirectly through upregulating NFIB (Fane et al., 2017); while MITF negatively regulates BRN2 via miR-211, a micro-RNA derived from the MITF-regulated gene melastatin (*TRPM1*) (Boyle et al., 2011). As a result, melanoma cells expressing low levels of MITF and high BRN2 (MITF^low^/BRN2^high^) are demonstrated to be significantly more tumorigenic than MITF^high^/BRN2^low^ cells when injected subcutaneously into mice (Goodall et al., 2008). In addition, BRN2 is shown to interact with the DNA damage response proteins PARP1 and Ku70/80 at sites of damage induced by UVB, chemotherapy or vemurafenib treatment, promoting error-prone repair via non-homologous end joining (NHEJ) and suppressing apoptosis (Herbert et al., 2019).

It’s therefore unsurprising given the role of BRN2 in driving invasion and metastasis that it has been similarly implicated in resistance to cell death by anoikis in melanoma. A recent study by our lab revealed that overexpression of BRN2 in human metastatic melanoma cell lines increased cellular viability when grown under non-adherent conditions (Pierce et al., 2020). The increased survival ability coincided with amplified expression of known markers of the anoikis-resistant and mesenchymal-like phenotypes; including the β1 integrin subunit (*ITGB1*), *TWIST1* and *MET*, as well as increased STAT3 phosphorylation. The study further demonstrated that inhibition of c-MET was able to significantly reduce percentage viability in BRN2 overexpressing cells in ultra-low attachment conditions.

Currently, there are no FDA registered inhibitors against BRN2, nor are there any in clinical trials. However, a study in neuroendocrine prostate cancer recently emerged detailing the development of a potent and specific small molecule inhibitor against wild-type BRN2 (B18-94) (Thaper et al., 2022) (Figure 4). Inhibition of BRN2 using B18-94, or inducible CRISPR/Cas9 knockout as a control, was shown to result in the upregulation of pathways for epithelial development and apoptosis, suggesting that direct inhibition of BRN2 may be a feasible way of inducing anoikis in cancer cells that overexpress BRN2. While yet to be examined in humans, *in vivo* administration of B18-94 via both intra-peritoneal (IP) and oral routes in a murine model suggests the compound has efficacious therapeutic likeness. Furthermore, treatment of xenograft tumours reduced tumour volume and cell proliferation, and increased apoptosis. Similarly, a synthetic peptide derived from the POU domain of BRN2 (R18H) has been demonstrated to induce apoptosis in B16-F10-Nex2 melanoma cells *in vitro,* while IP administration in C57BL/6 mice resulted in a significant reduction in the formation of metastatic nodules in the lungs, compared to untreated mice (da Cunha et al., 2019).

#### 2.4.2 STAT3

Signal transducer and activator of transcription 3 (STAT3) is activated downstream of receptor tyrosine kinase and growth factor receptors, particularly cMET/HGF, EGFR and PDGFR, as well as janus kinase (JAK) and Src Kinase pathways. Phosphorylation of STAT3 results in its dimerization and translocation to the nucleus where it promotes transcription. STAT3 has been extensively studied in the context of melanoma, where its constitutive activation is shown to promote melanoma cell proliferation, metastasis and invasion, and contribute to immune evasion by stimulating persistent expression of VEGF (Kortylewski et al., 2005; Lee et al., 2012; Swoboda et al., 2021; Graziani et al., 2022). Studies demonstrate that STAT3 drives melanoma cell survival through the upregulation of anti-apoptotic proteins Bcl-XL, Mcl-1, Cyclin D1 and survivin, and downregulation of p53 (Zhuang et al., 2007). As such, STAT3 has been identified as a contributor to the anoikis resistant phenotype in melanoma.

A study demonstrated increased STAT3 phosphorylation at Y705 in melanoma cell lines cultured in suspension conditions compared to adherent cells, driving upregulation of Bcl-2 and Mcl-1 (Fofaria and Srivastava, 2014). The subsequent increase in STAT3 activity increased migration and invasion of cells replated from suspension cultures *in vitro*, while knockout of STAT3 prevented the formation of tumours *in vivo*. An independent study confirmed these findings, revealing that p-STAT3 (Y705) stimulated anoikis resistance of B16-F10 melanoma cells as part of the FAK, p-ERK1/2 and PPARγ signalling pathways (Pei et al., 2017). Furthermore, STAT3 activity upregulated V-ATPase expression in B16-F10 cells to drive anoikis resistance, while pharmacological blockade of STAT3 repressed V-ATPase, inducing anoikis through ROS-mediated misfolded protein accumulation (Adeshakin et al., 2021b). An increase in STAT3 phosphorylation was similarly observed in melanoma cells cultured in ultra-low attachment conditions with an anoikis resistant phenotype induced by overexpression of BRN2 (Pierce et al., 2020).

The use of STAT3 inhibitors (STAT3i) for metastatic melanoma patients has been extensively investigated, given that combining STAT3i with anti-PD-1 immunotherapy has the potential to remodel the tumour microenvironment and resensitise treatment-refractory cells to vemurafenib treatment, while increasing CD8^+^ T cell infiltration into the tumour (Su et al., 2018; Zhao et al., 2020; Kim et al., 2022) (Figure 4). *In vitro* studies suggest the potential of utilising STAT3i to target anoikis resistance, with preclinical studies demonstrating that the synthetic inhibitor, AG-490, and the natural compound from black pepper, piplartine (PL), reduce anoikis resistance and induce PARP cleavage, while reducing the migratory potential of melanoma cells (Fofaria and Srivastava, 2014).

The FDA registered anti-helminthic drug niclosamide has potential inhibitory effects against STAT3. It’s unclear whether these effects are direct, however the molecule is predicted to bind to both the SH site and the Y705 phosphorylation site (Shi et al., 2017). Niclosamide may alternatively block STAT3 activation by targeting the androgen receptor variant V7 (AR-V7) upstream. Its use has been examined in phase I trials for castration resistant metastatic prostate cancer (NCT02532114) and refractory AML (NCT05188170). In melanoma, preclinical evidence revealed that niclosamide inhibited melanoma cell proliferation *in vitro,* independent of *BRAF* or *NRAS* mutation status, inhibiting tumour growth in xenograft models by uncoupling mitochondria and increasing metabolic stress (Figarola et al., 2018). Treatment of melanoma cells reduced STAT3 phosphorylation (Y705), inducing apoptosis via activation of Bax, reduction in Bcl-2 expression, and caspase-3 cleavage (Zhu Y. et al., 2019; Zheng et al., 2022). Niclosamide was further established to block melanoma cell migration and invasion, inhibiting activation of MMP-2 and 9, while inducing ROS generation in a dose-dependent manner. Other studies reveal improved delivery and efficacy of niclosamide against melanoma cells when the drug is packaged into liposomes (Hatamipour et al., 2019; Shah et al., 2022). A range of other STAT3 inhibitors have been examined in many cancers including melanoma, and are summarised in Table 3.

**TABLE 3:** STAT3 inhibitors.

Inhibitor	FDA registration year/trial phase	Clinical trial no.	Disease types	Evidence in melanoma
Napabucasin (BBI-608)	Phase III	NCT02753127, NCT01830621, NCT02178956	Colorectal and Gastric Cancer	Bitsch et al. (2022)
	Phase III	NCT02993731 (Sonbol et al., 2019)	Pancreatic Ductal Adenocarcinoma	
WP-1066	Phase I	NCT01904123	Recurrent Malignant Glioma and Metastatic Melanoma in the Brain	Kong et al. (2008), Hatiboglu et al. (2012), Tang et al. (2013), Sau et al. (2017)
	Phase I	NCT04334863	Medulloblastoma and Brain Metastases	
WP-1220 (MOL-4239)	Phase II	NCT01826201	Psoriasis	
		NCT04702503	Cutaneous T Cell Lymphoma	
NSC-741763	Phase I	NCT00696176 (Sen et al., 2012; Ramasamy et al., 2020)	Head and Neck Tumours	Niu et al. (1999)
SCV-07 (golotimod)	Phase II	NCT00968357	Chronic Hepatitis C	WO2012040656A2 (Tuthill and Sonis, 2012)
C188-9 (TTI-101)	Phase I	NCT03195699	Advanced Cancers, incl. Melanoma	
OPB-51602	Phase I	NCT01344876	Multiple Myeloma, Lymphoma, Leukaemia	
	Phase I	NCT01423903, NCT01867073	Advanced Cancer	
	Phase I	NCT01184807	Refractory Malignancies incl. Melanoma	Wong et al. (2015)
Danvatirsen (ISIS-481464, ISIS-STAT3Rx, AZD9150)	Phase I/II	NCT01563302 (Reilley et al., 2018), NCT03527147, NCT02549651	Advanced Cancers, Lymphoma, Diffuse Large B-Cell Lymphoma	
	Phase I	NCT01839604	Hepatocellular Carcinoma	
	Phase I/II	NCT02499328 (active)	Head and Neck Squamous Cell Carcinoma	
OPB-111077	Phase I	NCT01711034, NCT02250170, NCT01711034	Advanced Solid Tumours	
	Phase I	NCT03197714	Acute Myeloid Leukaemia	
SC-43	Phase I/II	NCT04733521 (recruiting)	Non-Small Cell Lung Cancer, Advanced Biliary Tract Cancer	
	Phase I	NCT03443622 (withdrawn)	Solid Tumours	
HL237	Phase I	NCT04633733, NCT03278470	Rheumatoid Arthritis	

Upstream, STAT3 is activated by the cytokine IL-6, and as such therapies blocking IL-6 are being investigated for their ability to prevent STAT3 activation (Table 4). In addition, as Janus kinases (JAK) are upstream mediators of STAT3 activation and signalling, a range of JAK inhibitors under investigation in clinical trials have demonstrated efficacy against melanoma in preclinical studies (Wu K. J. et al., 2017), and subsequently have the potential to be repurposed to block anoikis resistance by targeting JAK/STAT signalling in melanoma cells (Table 5).

**TABLE 4:** IL-6 inhibitors.

Inhibitor	FDA registration year/trial phase	Clinical trial no.	Disease types	Evidence in melanoma
Siltuximab (Sylvant, CNTO-328)	2014 (FDA)		Multicenteric Castelman’s Disease	Zaki et al. (2004)
	Phase I	NCT02641522	Type 1 Diabetes	
	Phase II Phase IIPhase I/II Phase II	NCT01484275, NCT00402181, NCT01531998, NCT04975555 (recruiting)	Multiple Myeloma	
	Phase II	NCT00433446	Prostate Cancer	
	Phase I/II	NCT00265135	Metastatic Renal Cell Carcinoma	
	Phase I/II	NCT04191421 (recruiting)	Pancreatic Cancer	
	Early Phase I	NCT05316116 (recruiting)	Granular Lymphocytic Leukaemia	
Bazedoxifene (Conbriza, Duavive, WAY-140424, TSE-424)	2013 (FDA)		Menopause	Chakraborty et al. (2021)
	Phase I/II Phase II	NCT02448771, NCT04821141 (recruiting)	Breast Cancer	
	Not Applicable	NCT04812808	Pancreatic Cancer	

**TABLE 5:** JAK inhibitors.

Inhibitor	FDA registration year/trial phase	Clinical trial no.	Disease types	Evidence in melanoma
Ruxolitinib (INCB018424, INC424)	2011 (FDA)		Myelofibrosis	Mo et al. (2022), Shen et al. (2022)
	Phase I/II Phase II	NCT02066532, NCT01594216	Breast Cancer	
	Phase I/II	NCT02155465	Lung Cancer	
	Phase II	NCT01423604	Pancreatic Cancer	
	Phase II	NCT01348490	Myeloproliferative Neoplasms	
Fedratinib (Inrebic, SAR302503, TG-101348)	2019 (FDA)		Myelofibrosis	
	Phase I	NCT01836705	Solid Tumours	
Pacritinib (ONX-0803, SB-1518)	Phase I	NCT02808455	Healthy Volunteers	
	Phase II	NCT04635059 (recruiting)	Prostate Cancer	
	Phase I/II	NCT04520269	Breast Cancer (with 1q21.3 copy number amplification)	
Tofacitinib (xeljanz, CP-690,550)	2012 (FDA)		Rheumatoid Arthritis	
	Phase III	NCT05326464 (recruiting)	Glioblastoma	
	Phase I	NCT04034238	Pancreatic Adenocarcinoma and Mesothelioma	
Itacitinib (INCB-039110)	Phase III	NCT03139604	Graft-versus-host Disease	
	Phase II	NCT01858883	Pancreatic Cancer and Solid Tumours	
	Phase I	NCT02646748 (Kirkwood et al., 2018; Beatty et al., 2019)	Solid Tumours incl. Melanoma	
	Phase I	NCT03272464 (active, not recruiting)	Melanoma	

#### 2.4.3 AP-1 family

The activating protein-1 (AP-1) family of transcription factors are comprised of homodimers between Jun proteins (cJun, JunB and JunD) or heterodimers between Jun and Fos (c-Fos, FosB, Fra1 and Fra2) (Wisdom, 1999; Eferl and Wagner, 2003; Gurzov et al., 2008), and demonstrate a wide range of functions that differ depending on the composition of the complex, the target gene, and the cell type, influencing cell growth, proliferation and cell cycle progression (Chinenov and Kerppola, 2001; Jin et al., 2011). AP-1 transcription factors act as part of the immediate-early response initiated by MAPK pathway signalling. As such, during melanomagenesis the constitutive activation of this pathway results in over-activation of the AP-1 factors, driving cellular dedifferentiation and transcriptional heterogeneity (Comandante-Lou et al., 2022). In particular, Fra-1 (encoded by the gene *FOSL1*) is important for melanoma progression as its accumulation triggers the transcription factor switch that drives (partial)-EMT, downregulating Zeb2 and *SNAI2* and directly upregulating *Zeb1* at its promoter (Caramel et al., 2013; Casalino et al., 2022). Subsequently, Fra-1 drives changes in cytoskeletal organisation, polarisation, motility and invasion (Dhillon and Tulchinsky, 2015; Casalino et al., 2022). Critically, Maurus and others demonstrated that *FOSL1* promotes anoikis resistant growth of melanoma cells on soft agar, and allows subcutaneous tumour growth *in vivo* via a Fra-1 target gene product, the chromatin modifier HMGA1 (Maurus et al., 2017).

Fra-1 was previously thought to be a non-viable target for pharmacological inhibition by small molecules, and as such most of the attempts to target the molecule focus on its destabilisation by inhibition of upstream kinases, or via targeting *FOSL1* mRNA (Sobolev et al., 2022). Some metabolites and molecules are reported to non-specifically inhibit its expression and function, such as PARP1 inhibitors, glucocorticoid dexamethasone, ranpirnase and rosiglitazone, while vemurfanib and retinoic acid are shown to activate Fra-1 expression (Zeng et al., 2022). However, a few polypeptide and small molecule inhibitors against Fra-1 have demonstrated promise in recent years (reviewed in (Casalino et al., 2022) Table 6 and Figure 4).

**TABLE 6:** AP-1 family inhibitors.

Target	Inhibitor	Type	Evidence in melanoma	References
c-Jun, Fos	TAM67	c-Jun dominant negative derivative peptide	Knocks down c-Jun in melanoma cell line from metastatic inguinal lymph node lesion	Madireddi et al. (2000)
			Blocked NFκB activation, downregulating genes for invasion and metastasis	Matthews et al. (2007)
			Reduced thymosin β4 in primary fibroblasts and melanoma cells	Nummela et al. (2006)
			Increased colony formation of primary and metastatic human melanoma cell lines in soft agar, induced vertical growth, loss of contact inhibition, morphology changes	Yang et al. (2004)
			Attenuated apoptotic effect of ATF2 derived peptide	Bhoumik et al. (2004)
AP-1, c-Fos	T-5224	Small molecule	Efficacy against triple-negative breast cancer cell lines, pituitary adenoma and human oral squamous cell carcinoma	Zhao et al. (2021), Kamide et al. (2016), Zhong et al. (2019)
			Induced PARP and caspase-3 cleavage in TERT-mutant melanoma cell lines	Liu et al. (2021b)

### 2.5 Other contributing factors

Additional factors, including the microenvironment and ECM, cellular metabolism, and production of reactive oxygen species (ROS) and nitric oxide (NO) are believed to contribute to anoikis resistance in melanoma. However, we have chosen to focus on the contribution and targeting of intracellular signalling cascades, and as such detailed discussion of these topics are beyond the scope of this review.

Research in the field of cellular microenvironment and ECM have focused on the contribution of MMPs and TIMP-1 to anoikis resistance. TIMP-1, despite being described as an MMP inhibitor, is interestingly shown to increase the survival of melanoma cells in suspension, and increase metastatic potential by interacting with β1 integrins, resulting in PDK1 activation (Zhu et al., 2001; Ricca et al., 2009; Toricelli et al., 2013; Toricelli et al., 2017). Furthermore, adhesion to fibronectin is thought to activate PI3K-Akt signalling, and protect cells from apoptosis (Boisvert-Adamo and Aplin, 2006; Fedorenko et al., 2016). Drugs targeting these processes have the potential to block anoikis resistance in melanoma cells [as reviewed in (Winer et al., 2018)].

Studies investigating the cellular metabolism of CTCs have identified autophagy as a contributing factor to anoikis resistance driven by p53, PI3K-Akt, mTOR, STAT and EGFR signalling (Guadamillas et al., 2011; Liu M. et al., 2021). The contribution of autophagy, as well as ferroptosis and necroptosis to anoikis resistance in melanoma were recently reviewed (Ashrafizadeh et al., 2019). Small molecule inhibitors targeting these pathways have the potential to induce anoikis in melanoma cells [as reviewed in (Wu et al., 2022)]. Regulators of fatty acid oxidation (FAO), CROT and CRAT, are shown to regulate anoikis in melanoma cells (Lasheras-Otero et al., 2022), while inhibitors of FAO, such as thioridazine and ranolazine may have therapeutic potential [as reviewed in (Munir et al., 2022)]. Anoikis induction is additionally linked to increased ROS and reduced NO production (Adeshakin et al., 2021b; Zhu et al., 2020; Monteiro et al., 2019; Ribeiro-Pereira et al., 2014; Giannoni et al., 2008; da Costa et al., 2018), and as such therapies that target these processes may be a viable option against melanoma cells (Adeshakin et al., 2021a). These topics were recently reviewed in (Sattari Fard et al., 2022).

## 3 Discussion/concluding remarks

There is an opportunity to repurpose existing therapies for patients with a high risk of disseminating melanoma. For instance, patients with ulcerated disease may benefit from novel therapy due to early dissemination leading to worse prognosis than those with non-ulcerated, but similarly staged melanoma. Further, treatments targeting pathways involved in invasion and cellular migration may prevent cells leaving the primary tumour, while those impacting anoikis resistance mechanisms may potentially target melanoma cells in circulation prior to the seeding of metastases (Figure 1). Ultimately, it is clear that transcriptomic testing is required to administer personalised targeted therapies based on the expression signature of an individuals’ tumour. This personalised approach will hopefully culminate in better health outcomes for patients with malignant melanoma.
